# Recent Advances in Micro- and Nano-Drug Delivery Systems Based on Natural and Synthetic Biomaterials

**DOI:** 10.3390/polym15234563

**Published:** 2023-11-28

**Authors:** Md. Harun-Or-Rashid, Most. Nazmin Aktar, Md. Sabbir Hossain, Nadia Sarkar, Md. Rezaul Islam, Md. Easin Arafat, Shukanta Bhowmik, Shin-ichi Yusa

**Affiliations:** 1 Department of Applied Chemistry, Graduate School of Engineering, University of Hyogo, 2167 Shosha, Himeji 671-2280, Hyogo, Japan; rashid685@diu.edu.bd (M.H.-O.-R.); nazmin29-846@diu.edu.bd (M.N.A.); shukantabhowmik@nstu.edu.bd (S.B.); 2 Department of Pharmacy, Faculty of Allied Health Sciences, Daffodil International University, Dhaka 1207, Bangladesh; sabbir29-1219@diu.edu.bd (M.S.H.); nadiasarkar181@gmail.com (N.S.); rezaul29-1301@diu.edu.bd (M.R.I.); easin29-1217@diu.edu.bd (M.E.A.)

**Keywords:** natural polymers, synthetic polymers, drug delivery, copolymers, biomimetic

## Abstract

Polymeric drug delivery technology, which allows for medicinal ingredients to enter a cell more easily, has advanced considerably in recent decades. Innovative medication delivery strategies use biodegradable and bio-reducible polymers, and progress in the field has been accelerated by future possible research applications. Natural polymers utilized in polymeric drug delivery systems include arginine, chitosan, dextrin, polysaccharides, poly(glycolic acid), poly(lactic acid), and hyaluronic acid. Additionally, poly(2-hydroxyethyl methacrylate), poly(*N*-isopropyl acrylamide), poly(ethylenimine), dendritic polymers, biodegradable polymers, and bioabsorbable polymers as well as biomimetic and bio-related polymeric systems and drug-free macromolecular therapies have been employed in polymeric drug delivery. Different synthetic and natural biomaterials are in the clinical phase to mitigate different diseases. Drug delivery methods using natural and synthetic polymers are becoming increasingly common in the pharmaceutical industry, with biocompatible and bio-related copolymers and dendrimers having helped cure cancer as drug delivery systems. This review discusses all the above components and how, by combining synthetic and biological approaches, micro- and nano-drug delivery systems can result in revolutionary polymeric drug and gene delivery devices.

## 1. Introduction

Nanomedicine uses nanotechnology to improve healthcare by manufacturing medication nanocarriers with the enhanced permeability and retention (EPR) effect. These nanocarriers may passively or actively target tumour tissues, resulting in more effective treatment. In the past two decades, pharmaceutical research and development has focused on developing drug delivery vehicles. A better drug transporter is biocompatible, biodegradable, non-toxic, and delivers active substances to the action site. Until recently, liposomes, organic and inorganic nanoparticles, and hydrogels were considered promising drug carriers [1,2]. Due to their abundance in nature and unique properties, including sustainability, biocompatibility, and biodegradability, natural biopolymers are gaining favour in drug delivery systems (DDSs) [3,4]. Living cells build natural biopolymers by covalently bonding monomeric units to form large molecular-weight molecules. Polysaccharides and proteins are the most common natural biopolymers, which include chitosan, cellulose, dextran, starch, pectin, collagen, gelatin, fibronectin, elastin, keratin, actin, myosin, etc. Polymers made up of long chains of amino acid residues (proteins) exhibit a broad range of physical and chemical characteristics. Oligopeptides are linear chains of 20–30 amino acids. Nondegradable materials used in biomedical applications can be replaced by synthetic biodegradable polymers. The majority of synthetic biodegradable polymers used today as common materials and even in the biomedical industry belong to the polyester family. In most cases, poly(glycolide), poly(lactide), poly(caprolactone), and their copolymers are part of applications and research. Various synthetic polymers based on poly(amino ester) (PAE) are suggested as candidates for gene and drug delivery owing to their pH responsiveness, which contributes to efficient delivery performance [5].

This review addresses natural and manmade/synthetic polymers, their desirable qualities, ongoing clinical trials, and their limitations in regard to drug delivery systems. Moreover, we discuss how combining two or more biomaterials with enhanced capabilities, such as copolymers, polymer–polymer blends, or composites, may meet most therapeutic needs. The existing literature is reviewed, and a scaffold design, commercial viability, and manufacturing methods are also examined.

## 2. Data Source and Search Strategy

We comprehensively searched online publications in the WOSCC (Web of Science Core Collection) database on 25 September 2023. The publication timeframe ranged from an unspecified date to 25 September 2023. To prevent bias stemming from daily database updates, we specifically looked into articles on a single day. Our focus was on English-language publications, with only “articles” and “reviews” being considered. Prior to assessing the relevance of the literature to the theme of natural and synthetic biomaterials in drug delivery as well as ongoing clinical research, we eliminated publications that did not meet the specified language and article-type criteria by evaluating their titles and abstracts.

## 3. Nano-Based Drug Delivery Systems

### 3.1. Fundamentals of Nanotechnology-Based Drug Design Methodologies

Nanomedicine is a subspecialty of medicine that makes use of nanoscale materials, such as biocompatible nanoparticles [6] and nanorobots [7], to carry out a variety of biological tasks, such as diagnostics [8], transportation [9], sensing [10], and actuation [11]. Before the conventional method of formulating vaccinations was established, medicines with very low aqueous solubility presented a number of difficulties in terms of biopharmaceutical delivery. These difficulties included restricted bio-access on oral intake, a lower diffusion capacity into the outer membrane, larger intravenous (IV) dosage requirements, immunogenicity, and unwanted side effects. It is possible that if nanotechnology was included in the pharmaceutical distribution process, all of these problems would be eliminated. Drug design at the nanoscale has been the subject of extensive research and is currently the most cutting-edge technology in the field of nanoparticle applications. This is due to the fact that nanoscale drug design offers an abundance of benefits, including the capability to modify properties such as solubility, drug release profiles, diffusivity, bioavailability, and immunogenicity. Because of this, it may be feasible to devise more comfortable administration techniques that have less toxicity, fewer side effects, better biodistribution, and a longer pharmaceutical life cycle [12].

DDSs were developed to either direct therapeutic chemicals to a particular location in a more concentrated fashion or to disperse therapeutic chemicals to a certain area in a more manageable way. Self-assembly is defined as the process through which the assembly of components results in the spontaneous emergence of well-defined forms or patterns [13]. Endocytosis and absorption via a system of mononuclear phagocytes are also very important [14]. As a result of the hydrophobic properties of a structure, medicines may be injected into an internal cavity. When nanostructure components are guided to specific locations, the amount of medication that is expected to be released is achieved despite the low concentration of the medicine that is kept in a hydrophobic environment. Both passive and active methods of medication administration are viable options for nanostructured DDSs. As a result of the hydrophobic nature of drugs, they are often extensively absorbed into the interior cavity of a structure. When nanostructure materials are directed to certain locations, pharmaceuticals are kept in an environment that is hydrophobic, and the appropriate quantity of medication is released [14]. To facilitate distribution more easily, supplied medications are conjugated to the transporter nanostructure material as quickly as is practically possible. If a medication is released from its nanocarrier system at the incorrect moment, it will not reach the target it was designed for, and it will rapidly dissociate from the carrier, resulting in a reduction in its bioactivity and effectiveness [15]. 

Another essential feature of a DDS is drug targeting, wherein nanoparticles or nano-formulations make up the DDS and are split up into active and passive categories. In order to successfully target antibodies and peptides to receptor complexes expressed in a particular area, several DDSs are coupled with antibodies and peptides. Active targeting involves the use of antibodies and peptides in combination with pharmaceutical delivery methods to connect to receptor structures that are expressed in the target site. The produced drug carrier complex is directed to the target site by affinity or binding, which is governed by pH, temperature, molecular size, and shape. This process takes place while the complex travels through circulation. The receptors that are located on the cell membranes, the lipid components that are located on the cell membranes, and the antigens or proteins that are located on the surface of the cells are the primary targets in the body [16]. The majority of nanotechnology-assisted DDSs are now under investigation for use in the treatment and prevention of cancer.

### 3.2. Diagnostic, Detection, and Imaging Applications of Biopolymeric Nanoparticles

The combination of treatment and diagnosis is known as theragnostic therapy, and it is used widely in cancer treatment [17]. When used in theragnostic therapy, nanoparticles have the potential to improve the disease diagnosis, treatment localization, stage, and treatment response. In addition, nanoparticles have the capability of transporting tumour-targeted therapeutic medication, which may then be released into the body in response to either an internal or external stimulus [18]. Chitosan, also known as chitin, is a biomaterial that has distinctive qualities, such as biological uses and functional groups [19]. Chitosan is being employed to encapsulate or coat a broad variety of nanoparticles, which will result in a diversity of particles with various activities that may be used in the detection and diagnosis of illness [20]. Using oleic acid-coated iron oxide nanoparticles encapsulated in oleic acid-conjugated chitosan (oleyl-chitosan), Lee et al. [18] investigated the accretion of nanoparticles in tumour cells via the EPR effect in vivo for analytical reasons. This was done using oleic-acid-coated iron oxide nanoparticles for magnetic resonance imaging (MRI). In vivo, experiments of cyanine-5-attached oleyl-chitosan nanoparticles demonstrated high signal intensity and recovery in tumour tissues using both techniques. 

Yang et al. [19] reported nanoparticles with improved 5-aminolevulinic (5-ALA) release in the cell lysosome; the nanoparticles were produced by physically connecting alginate to folic acid-modified chitosan. The nanoparticles were extremely effective for light-mediated colon cancer (CC) cell detection. According to the results, the modified nanoparticles were readily endocytosed by CC cells via a folate receptor-based endocytosis mechanism. Due to the usage of deprotonated alginate, there was a reduction in the binding strength between 5-ALA and chitosan. As a consequence, the charged 5-ALA was delivered to the lysosome, which led to an accumulation of protoporphyrin IX for photodynamic detection inside the cells. Researchers found that chitosan-based nanoparticles coupled with alginate and folic acid are good vectors for delivering 5-ALA to CC cells while also allowing for endoscopic fluorescence monitoring. 

Cathepsin B (CB) is an enzyme that plays an essential role in the detection of metastasis because of its close connection to the metastatic process and its prevalence in the pericellular regions where it takes place. Ryu et al. [20] produced fluorogenic peptide and tumour-targeting glycol chitosan nanoparticles, which were incorporated on the surface of a CB-sensitive nanoprobe. The resulting nanoprobe was spherical, with a diameter of 280 nm, and did not glow when placed in biological environments. In three different rat metastatic models, a CB-sensitive nanoprobe was used in conjunction with non-invasive imaging to differentiate between metastatic and healthy cells. 

Another example of a biopolymeric molecule is hyaluronic acid (HA). This glycosaminoglycan, which is biocompatible and has a negatively charged ion, is found in the extracellular matrix [21]. As a result of the interaction between the receptor and the linker, HA has the potential to bind to the CD44 receptor, which is overexpressed in certain cancer cells. As a consequence of this, HA-modified nanoparticles have the potential to assist in the diagnosis and treatment of cancer [22,23,24]. Dopamine-modified HA was used to coat the surface of iron oxide nanoparticles in a study carried out by Wang and co-workers [25]. Siyue et al. [26] created HA nanoparticles with varying diameters using varying degrees of hydrophobic HA replacement. Nanoparticles were given to animals afflicted with malignancies, and the treatment responses were analyzed. For the purpose of the early detection and targeted treatment of CC, the same research group developed a potent and flexible thermostatic system based on PEG-conjugated HA nanoparticles (P-HA-NPs). To determine whether or not the nanoparticles were effective against cancer, they were first chemically coupled to a near-fluorescent dye, cyanine 5.5 (Cy5.5), and then encapsulated with the anticancer medication irinotecan (IRT). The therapeutic potential of P-HA-containing nanoparticles was then investigated using a variety of CC animal models. The near-infrared fluorescence imaging technology was successfully used to scan tiny and early-stage malignancies as well as CCs that were implanted in the liver. This was accomplished after an IV injection of fluorescent dye-associated nanoparticles (Cy5.5-P-HA-NPs). The remarkable ability of drug-containing nanoparticles (IRT-P-HA-NP) to target tumours resulted in a considerable reduction in the growth of malignancies while causing no damage to the body as a whole. Cy5.5-P-HA-NPs are a potential tool for concurrently analysing many aspects of the healing process [27].

The natural polymer alginate, which is obtained from brown seaweed, has been subjected to intensive research for the purpose of determining whether or not it could be used in the medical field. This is due to the fact that alginate possesses a number of desirable qualities, such as low toxicity, compatibility, and an easy gelling ability, when divalent cations are present. Baghbani et al. [28] employed alginate-stabilized perfluorohexane (PFH) nanodroplets to administer doxorubicin (DOX). In addition to determining its potential therapeutic utility, they subsequently examined the sensitivity of the nanodroplets to ultrasonography and imaging. Treatment with ultrasound-assisted therapy using DOX-loaded PFH nanodroplets also showed significant promise in breast cancer rat models. The degree to which the tumour was able to be broken down was an indicator of how well the treatment worked. Podgórna et al. [29] produced gadolinium nanogels (GdNGs) for MRI scanning and the loading of hydrophilic pharmaceuticals. The diameter of the gadolinium alginate nanogels was an average of 110 nm, and their stability lasted for sixty days. In MRI imaging, gadolinium combinations are often used as positive contrast agents due to the inherent paramagnetic characteristics of gadolinium. The spin-lattice relaxation time (*T*_1_) was dramatically shortened due to the presence of GdNGs in comparison to controls. As a consequence of this, alginate nanogels have the potential to be used in the medical industry as contrast enhancers.

It is believed that dextran, a non-toxic neutral polymer, was the first exopolysaccharide used in medicine by bacteria. Dextran is a peculiar substance since it is safe for human consumption, does not cause any harm, and can be broken down by biological processes. The cancer treatment known as photodynamic therapy eliminates cancer cells while sparing the surrounding healthy tissue from damage. Ding et al. [30] built a nanoparticulate multifunctional composite system for near-infrared (NIR) imaging and MRI. They did this by encapsulating Fe_3_O_4_ nanoparticles in dextran nanoparticles and then coupling the dextran nanoparticles to redox-responsive chlorine 6 (C6). The redox biological reaction that occurs as a result of the nanoparticles generates a fluorescent signal that has an “on/off” characteristic, which enables accurate tumour imaging. In addition, improvements in magnetic targeting capabilities led to an increase in the effectiveness of photodynamic therapy, both in vitro and in vivo. The production of theragnostic nanoparticles and glioma cells was carried out by Ali et al. [31] using C6 mice. In order to produce these particles, gadolinium oxide nanoparticles were first coated with either paclitaxel (PTX) or folic acid (FA)-conjugated dextran. The MTT test was used to evaluate both the chemotherapeutic effects of PTX on C6 glioma cells and the bioprotective advantages of a dextran coating. Due to the paramagnetic properties of the gadolinium nanoparticles, the nanoparticles were able to penetrate C6 tumour cells through a process known as receptor-mediated endocytosis. Additionally, the nanoparticles displayed enhanced contrast MRI concentration-dependent activity. When it came to inhibiting cell growth, uncoated gadolinium nanoparticles were shown to be less efficient than their multifunctional counterparts. As a consequence of this, drugs that are paramagnetic and chemotherapeutic may be produced by utilizing theragnostic nanoparticles containing both FA and PTX.

## 4. Drug Delivery Using Synthetic Polymers

### 4.1. Poly(2-hydroxyethyl methacrylate)

**Figure 1:**
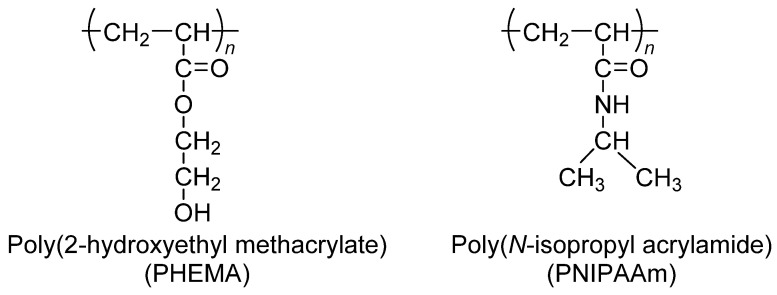
Chemical structure of poly(2-hydroxyethyl methacrylate) (PHEMA) and poly(*N*-isopropyl acrylamide) (PNIPAAm).

Poly(2-hydroxyethyl methacrylate) (PHEMA) hydrogel for intraocular lens components was synthesized via solution polymerization using 2-hydroxyethyl methacrylate (HEMA) with cross-linking agents, such as ethyleneglycoldimethacrylate and triethyleneglycoldimethacrylate [32]. In cancer research, PHEMA (Figure 1) is often used to wrap cell-culture flasks to reduce the amount of cell adhesion and increase the creation of spheroids. There are two older alternatives to PHEMA: agar and agarose gels [33]. For the purpose of developing drug delivery systems, equilibrium swelling, structural characterization, and solute transports in swollen PHEMA gels cross-linked with tripropyleneglycol diacrylate (TPGDA) were investigated across a large TPGDA concentration range [34]. In order to get a better understanding of the mechanism of drug–polymer interaction and its influence on the drug-release behaviour of controlled-release polymeric devices, the physical and chemical features of pilocarpine extracted from PHEMA hydrogels were investigated. PHEMA hydrogels are often employed in the field of biomedical implants. PHEMA, which is biocompatible, is also a potential coating for ventricular catheters [35,36] because of its high hydrophilicity, which provides resistance to protein fouling.

### 4.2. Poly(N-isopropyl acrylamide)

Poly(*N*-isopropyl acrylamide) (PNIPAAm) dissolves in water at a low temperature; however, it cannot dissolve in high-temperature water (Figure 1). In the 1960s, temperature-sensitive polymers were a prevalent research subject [37]. The lower critical solution temperature (LCST) of thermo-sensitive PNIPAAm was 32 °C. To determine the thermodynamic properties of the system, the phase diagram and amount of heat absorbed during phase separation were used in the calculation [38]. While carrying out radical polymerization of *N*-isopropyl acrylamide (NIPAAm), it is common practice to make use of the initiator known as azobis(isobutyronitrile). Thermo-responsive polymers have a variety of applications in biological and medical fields, including medicine and gene transfer [39]. An investigation was conducted into the temperature dependence of the swelling of cross-linked poly(*N*,*N*-alkyl substituted acrylamides) in water. Thermo-sensitivity of water swelling has been linked to the delicate balance of hydrophilic and hydrophobic groups on polymer chains, which is determined by the size, conformation, and mobility of alkyl side-chain groups [40]. This balance is regulated by the size of the alkyl side-chain groups. This method has been used to create hydrophilic and hydrophobic coatings on a cell culture surface of PNIPAAm-grafted polymers in a reversible manner [41].

NIPAAm was copolymerized with acrylic acid (AAc) to develop temperature- and pH-sensitive hydrogels. The influence of polyelectrolytes on the LCST of temperature/pH-sensitive hydrogels was investigated within the pH range of the swelling ratio. In the same conditions and in the presence of poly(allyl amine) (PAA) as a polyelectrolyte, an investigation into the swelling ratio of hydrogels was carried out [42]. Some of the discussed subjects in the study were pH, redox responsiveness, hypoxia sensitivity, and other tumour microenvironmental-sensitive nanoparticle in situ stimuli.

### 4.3. Poly(ethylenimine)

**Figure 2:**
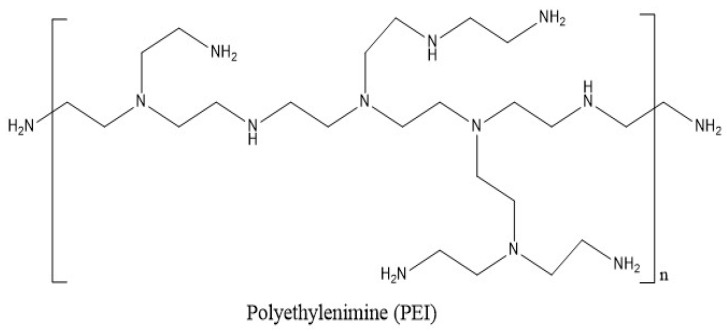
Chemical structure of branched poly(ethylenimine) (BPEI) for polymeric drug delivery.

When the pH is low, the compound known as poly(ethylenimine) (PEI) is soluble in hot water, ethanol, and chloroform. The compound is impervious to the solubility of cold water, acetone, benzene, and ethyl ether. Branched PEI (BPEI) is a polymer with repeating units composed of ethylene diamine groups. Aziridine ring-opening polymerization led to the formation of BPEI. BPEI is a cationic polymer that contains primary, secondary, and tertiary amino groups (Figure 2). Such types of water-soluble polymers having a high density of amines are one of the most promising cationic vectors for gene delivery. Hence, constructing nanocarriers that contain PEI has attracted considerable research efforts in gene therapy because of the synergetic effects of PEI molecules (for their efficient transfection) and the multi-functionality of nanoparticles in delivery. BPEI can be prepared with a divergent synthesis method from an ethylenediamine core. Day by day, highly branched polymers have also attracted growing interest in various fields, especially in drug delivery, gene delivery, and diagnosis [43,44]. Major applications of BPEI include the stable combination with other positively charged particles, layer-by-layer construction of nanoparticle surfaces, binding to negatively charged substrates or larger particles, and colour engineering.

### 4.4. Dendritic Polymers

**Figure 3:**
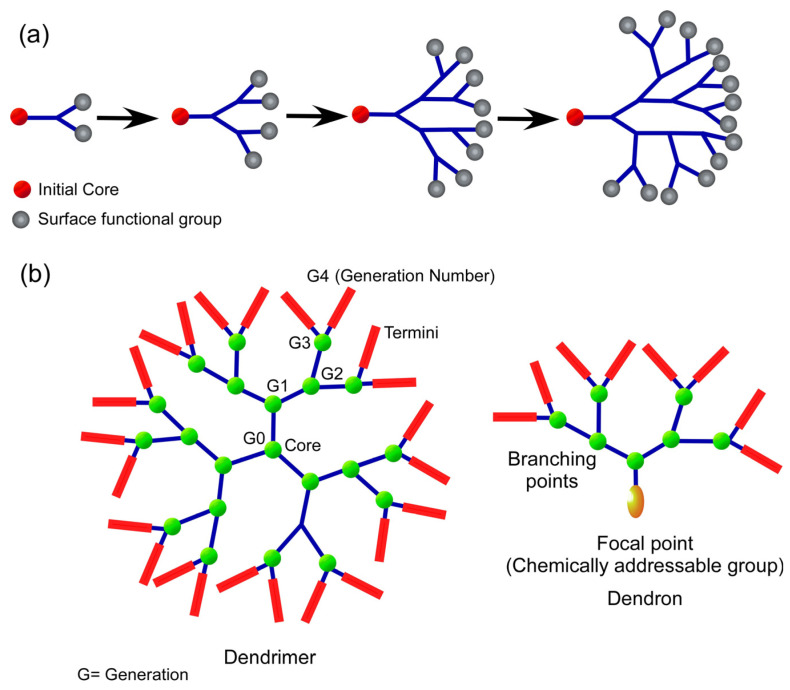
(**a**) Schematic design for divergent dendrimer manufacturing for drug delivery; (**b**) drug delivery dendrimer and dendron chemical structures.

Dendrimers are three-dimensional structures that have a high bifurcation level, are monodisperse, and have clear boundaries. Effective drug delivery systems may be characterized by their spherical form, which makes it possible for them to be quickly functionalized in a regulated manner [45]. The path to dendrimer formation can be convergent (the dendrimer develops inwards from the exterior) or divergent (the dendrimer expands outwards from its centre) [46]. Dendritic polymers are distinguished by their large population of terminal functional groups as well as their low solution or melt viscosity and great solubility (Figure 3). The scale of dendritic polymer synthesis processes may be regulated and varied, as can their branching and overall utility.

Dendrimers are a kind of polymer that is classified under the dendritic polymer family. Other types of polymers included in this family are linear, cross-linked, and branched polymers. The design and production of biocompatible dendrimers, in addition to their use in a range of bioscience sectors that include drug delivery, immunology, and vaccine generation, have been the primary focus of dendrimer research [47,48]. Dendrimers are a form of reducible polymer that has also been investigated for their potential to effectively transport genes [49].

### 4.5. Biodegradable and Bioabsorbable Polymers

**Figure 4:**
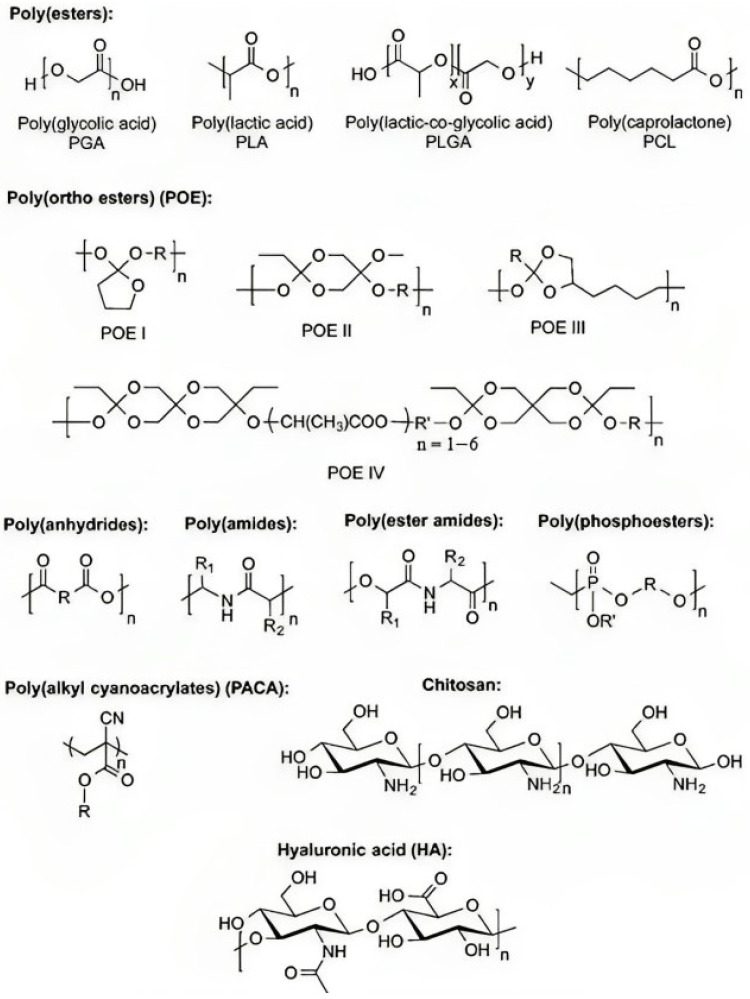
Biodegradable polymers for polymeric drug delivery with typical monomer units (Reproduced from [51]).

If an implant is only going to be needed for a short amount of time, the best choice for drug carriers would be bio-absorbable drug delivery devices [50]. Aliphatic polyesters are examples of synthetic biodegradable polymers. Some examples of aliphatic polyesters are PGA and PLA. PCL and polydioxanone are the polymers used as bio-absorbable drug delivery devices the majority of the time. Many other types of polymers have been developed, including polyesters, poly(ortho ester)s, polyanhydrides, and biodegradable polycarbonates (Figure 4) [51,52]. There are several types of biodegradable polymers, including hydroxy acid, polyanhydride, polyamide, poly (ester amide), polyphosphoester, poly(alkyl cyanoacrylate), PHA, and natural sugars such as chitosan. In drug delivery systems, synthetic biodegradable polymers are favoured over natural biodegradable polymers [53,54,55]. This is due to the fact that natural biodegradable polymers are immunogenic.

## 5. Drug Delivery Methods Using Nanoparticles

### 5.1. Polymeric Micelles

Polymer-based micelles are nanostructures composed of amphiphilic block copolymers that may self-assemble into a core-shell structure when placed in an aqueous solution. While the hydrophilic shell renders the whole system water soluble and stabilizes the core, the hydrophobic core may include hydrophobic medications, such as camptothecin, docetaxel, and PTX. These nanostructures have a promising future in the field of hydrophobic drug delivery because their interior core shape makes it possible for drugs to be absorbed, which results in higher stability and bioavailability [56].

The production of polymeric micelles may be accomplished by direct polymer dissolution in a solvent, solvent evaporation, and dialysis methods [57]. Micelle creation is affected by a number of factors, including the size of the hydrophobic chain on the amphiphilic molecule, the concentration of amphiphiles, the temperature, and the kind of solvent system [58]. The process of micelle creation starts when the concentration of amphiphilic molecules reaches a critical level, which is also referred to as the critical micelle concentration (CMC). Because of their diminutive size, amphiphilic molecules are only capable of existing on their own in trace quantities [59]. During direct dissolution, the copolymer and pharmaceuticals interact with one another on their own in an aqueous environment, resulting in a drug that is packed with micelles. In the method known as solvent evaporation, the needed medication and the copolymer are first dissolved in a volatile organic solvent. Next, the drug in the solution and the copolymer in the organic solvent are mixed in a dialysis bag and dialyzed, making use of micelle formation [60].

**Figure 5:**
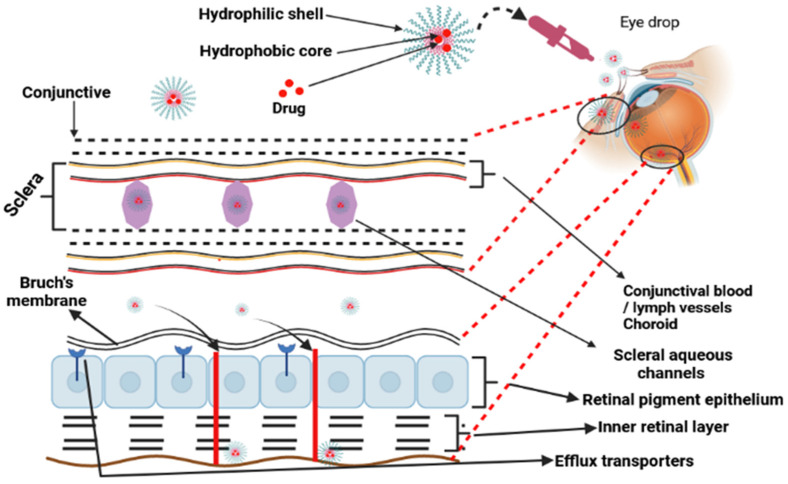
Polymeric micelles employed to reach posterior ocular tissues via the transscleral channel following topical treatment.

Stimuli that promote penetrability and the hold effect include monoclonal antibodies connected to the corona of the micelle or a carefully targeted ligand molecule complexed to the surface of the micelle [61]. The use of polymeric micelles may be beneficial when it comes to the administration of cancer treatments [58] and the delivery of eye medications (Figure 5) [62]. For the treatment of progressive vitreoretinopathy, polymeric micelles were encased in nanoparticles produced using the micellization of poly(ethylene glycol)-*block*-poly(bisphenol A carbonate). ARPE-19 cells were not affected by the cytotoxicity of the nanoparticles, which had a diameter of 55 nm. When compared to free medicines, the micellar formulation demonstrated a significant increase in the ability to suppress cell growth, adhesion, and translocation [63]. After appropriate treatments are administered, polymeric micelles are commonly injected into the posterior ocular tissues via the transscleral channel [61].

### 5.2. Dendrimers

Dendrimers for oral medication delivery have received the most attention because they are water soluble and can pass through epithelial tissue [64]. Because dendrimers include amine groups, their potential as medicinal agents are rather restricted. The cationic or negatively charged nature of dendrimers results in them often being changed in an effort to lessen or remove their toxicity. The following is a list of procedures used to load medicines into dendrimers: encapsulation, electrostatic contact, and covalent conjugation, which are all included in simple encapsulation [65]. Dendrimers are primarily responsible for the delivery of drugs through two different pathways: (a) in vivo degradation of the covalent bonding of the drug dendrimer as a result of suitable enzymes or a favorable environment that can cleave the bonds and (b) drug discharge as a result of changes in the physical environment, such as pH, temperature, and so on. Dendrimers have the potential for use in-drug administration through transdermal, oral, ophthalmic, pulmonary, and targeted methods.

Jain et al. [66] showed that folate-attached poly(*L*-lysine) dendrimer-encapsulated DOX is a promising cancer prevention drug carrier model due to its pH-dependent drug discharge, target selectivity, antiangiogenic ability, and anticancer potential. The use of DOX-folate conjugated poly(*L*-lysine) dendrimers resulted in a 121.5% increase in DOX concentration in the tumour. Using folate-conjugated polypropylene imine dendrimers, Kaur et al. [67] developed a pH-sensitive methotrexate (MTX) nanocarrier for cancer cell targeting and anticancer therapy via folate-conjugated poly(propylene imine) (PPI) dendrimers (FAPPI). FAPPI is considered a pH-sensitive DDS. In vitro studies with MCF-7 cell lines demonstrated substantial release, enhanced cell uptake, and mild cytotoxicity [68]. In addition to these results, the generated formulations, which were methotrexate MTX-loaded and folic acid-conjugated generation 5 PPI, were preferentially taken up by tumour cells compared to the free drug.

### 5.3. Inorganic Nanoparticles

Inorganic nanoparticles include gold, silver, iron oxide, and silica nanoparticles. A very small number of inorganic nanoparticles have been approved for use in therapeutic applications, but the vast majority are currently undergoing testing in clinical studies. Surface plasmon resonance (SPR) is only found in silver and gold nanoparticles; liposomes, dendrimers, and micelles do not possess this property. Among inorganic particles, gold and silver nanoparticles provide a wide range of advantages, the most notable of which is their excellent biocompatibility and flexible surface functionalization. There is very little evidence to suggest that paracellular transport and transcytosis really take place in living organisms [68] despite the fact that these processes have been postulated. Although paracellular and transcytosis transport and absorption have been proposed as possibilities, little is known for certain about how these processes work in the body. The surfaces of gold nanoparticles may have drugs conjugated to them through ionic or covalent bonding as well as through physical absorption. These drugs can then be delivered and controlled through the use of biological stimuli or light activation [69]. In recent years [70], the use of metallic nanoparticles has become more common in a variety of medical applications, including bioimaging, biosensors, target/sustained drug delivery, hyperthermia, and photoablation. By adding functional groups, these nanoparticles have the potential to interact with antibodies, medicines, and other ligands, hence increasing their value for use in biological applications [71]. Zinc oxide, titanium oxide, platinum, selenium, gadolinium, palladium, and cerium dioxide nanoparticles are also all gaining increased interest.

In order to facilitate the release of ornidazole, Prusty and Swain [72] developed an interconnected and spongy polyacrylamide/dextran nano-hydrogel hybrid system. This system had covalently bonded silver nanoparticles and obtained a 98.5% success rate in vitro. Another study used laser pyrolysis to create iron oxide nanoparticles, which were then coated with violamycine B1 and antracyclinic antibiotics [73]. These nanoparticles were tested in MCF-7 cells for cytotoxicity and anti-proliferation properties and compared to commercially available iron oxide nanoparticles.

### 5.4. Nanocrystals

Nanocrystals are defined as pure solid pharmaceutical particles with dimensions of fewer than one thousand nanometres. The performance of nanocrystal suspensions in thin liquid media may be significantly enhanced by the use of a surfactant component known as nano-suspension. Water, or other aqueous or non-aqueous media, such as liquid PEG and oils, are used as the dispersion mechanism [74]. Nanocrystals offer unique properties, such as enhanced saturation solubility, speedier dissolution, and improved adherence to surface and cell membranes. Both bottom-up and top-down methods used to produce nanocrystals are examined here. Sono-crystallization, precipitation, high gravity-controlled precipitation technology, multi-inlet vortex mixing methods, and the limited impinging liquid jet precipitation technique are all components of the top-down approach [75]. This method is somewhat pricey since it requires the use of an organic solvent that is subsequently thrown away. Grinding and homogenization steps take place under intense pressure during the bottom-up process. Milling, high-pressure homogenization, and precipitation are the three processes most often used when attempting to create nanocrystals. Nanocrystals increase medication absorption by increasing solubility, suspension rate, and the ability to keep the intestinal wall in place. Cinaciguat nanocrystals coated in chitosan microparticles were the vehicles that Ni et al. [76] used to deliver a hydrophobic medication to the lungs. Swelling and muco-adhesive properties of the polymers were used in the production of nanoparticles for continuous medication release. They found that sickness may limit the effectiveness of inhalation, which suggests that more research is required to prove if this method is promising [77].

### 5.5. Quantum Dots

Quantum dots (QDs) are semiconductor nanocrystals that range in diameter from 2 to 10 nm and feature size-dependent optical characteristics that include absorbance and photoluminescence [78]. QDs have attracted a lot of interest in the field of nanomedicine since they emit in the NIR region (650 nm), which is a particularly desirable property in biomedical imaging due to decreased tissue absorption and light scattering at this wavelength [79]. Biocompatible multifunctional graphene oxide QDs with a brilliant magnetic nanoplatform were created by Shi et al. [79] for detecting and identifying individual liver cancer tumour cells (glypican-3-expressing Hep G2). The researchers claim that by combining an anti-glypican-3 antibody with a nanoplatform, they are able to selectively eliminate Hep G2 hepatocellular cancer cells from contaminated blood samples. Coating QD antibodies with norbornene-displaying polyimidazole ligands was the method that Ahmad et al. [80] used to develop a new fluorophore for intravital cytometric imaging. This fluorophore was utilized to highlight hematopoietic stem and progenitor cells in bone marrow in vivo. Additionally, a single light source has the potential to excite QDs of various sizes and/or compositions, which may lead to a wide spectrum of colour emission [81]. In terms of multiplex imaging, we feel that QDs have a very bright future. In the field of medicine, QDs have been the subject of substantial research as potential targets for targeted medication administration, sensors, and bioimaging. Recent years have seen a proliferation of studies on the use of QDs as contrast agents in in vivo imaging [82]. 

As a parenteral multifunctional system, Olerile et al. [83] created a theragnostic system based on co-loaded QDs and anti-cancer drugs in nanostructured lipid carriers. This system is intended to be administered intravenously. A tumour growth suppression rate of 77.9% and an encapsulating efficacy of 80.7% were achieved by the spherical nanoparticles. According to the findings of the study, the procedure has the potential to be utilized to target and detect H22 tumour cells. Cai et al. [84] created pH-responsive ZnO QDs coated with PEG and HA acid. These QDs were stable in physiological environments and targeted cells that expressed the HA receptor CD44. This nanocarrier was used to investigate both short-term and long-term DOX release. The physiological pH was used to load DOX into the nanocarrier by either combining it with Zn^2+^ ions or conjugating it to PEG. The appearance of DOX in tumour cells only occurred after the ZnO QDs had been degraded in an acidic environment inside the cell. The researchers found that increasing the amount of DOX and ZnO QDs in treatment increased its anticancer effect.

### 5.6. Protein and Carbohydrate Nanoparticles

**Figure 6:**
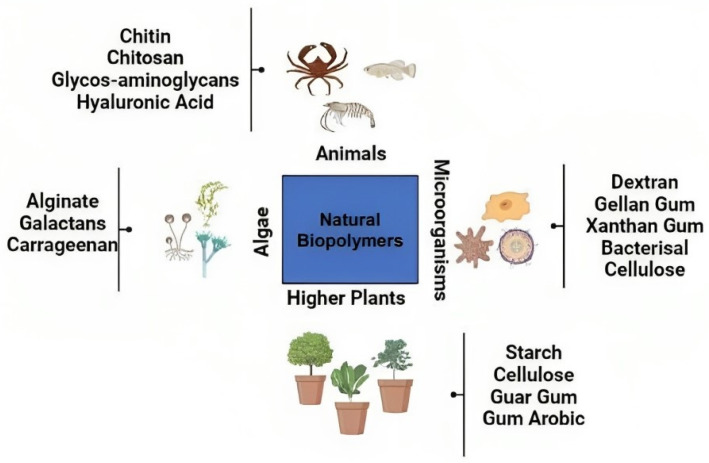
Natural biopolymers for nanomedicine applications from various sources. Natural biopolymers could come from higher plants, animals, microorganisms, and algae.

The term “natural biopolymers” refers to polysaccharides and proteins that originate chitosan from biological sources, such as plants, animals, and microbes [85]. Protein-based nanoparticles are an attractive choice due to their ability to metabolize drugs and other ligands as well as their biodegradability and ease of functionalization for drug attachment. In order to produce natural biopolymers, water-soluble proteins, such as bovine and human serum albumin, and insoluble proteins, such as zein and gliadin, are used [86]. Coacervation and desolvation, emulsion and solvent extraction, complicated coacervation, and electro-spraying are the industrial processes that are most often used to create such particles. The targeting mechanism of protein nanoparticles may be strengthened and improved by including targeting ligands. Targeting ligands are identifiers for certain cell and tissue types via a process known as chemical modification [85]. Polysaccharides, also known as polycarbohydrates, are the most abundant carbohydrates found in food; polysaccharides are constructed from sugar units linked to one another by *O*-glycosidic bonds. Polysaccharides have the potential to exhibit a broad variety of physical and chemical properties depending on their monomer composition and biological source [86]. One of the most significant difficulties of using polysaccharides in nanomedicine is the fact that these molecules are prone to oxidation (degradation) at high temperatures (far over their melting point), which are often necessary in industrial procedures. In addition, the vast majority of polysaccharides are water soluble, which restricts their applicability in nanomedicine applications, such as tissue engineering [87]. In aqueous environments, polymer chain crosslinking has been shown to be an effective method for ensuring the structural integrity of the polysaccharide chain. The many different sources of polysaccharides that have been exploited in nanomedicine are shown in Figure 6. These biopolymers are used in nanomedicine and drug delivery due to their adaptability and specific properties. These properties include their ability to originate from soft gels, flexible fibers, and hard shapes, which can make them porous or non-porous, and their high similarity to components of the extracellular matrix, which may help them avoid immunological reactions [88]. Although not much research has been done on these nanoparticles, the fact that they are made of biocompatible materials indicates that there is a significant amount of promise for their use in future drug delivery techniques. Bovine serum albumin was artificially produced by Yu et al. [89], who then studied the capacity of the protein to bind to and infiltrate the cochlea and middle ear of guinea pigs. They have also looked into the loading capacity and release behaviours of nanoparticles that have the potential to be used as drug transporters. Their goal was to determine whether or not these nanoparticles could provide greater bio-suitability, increased drug loading capacity, and a well-ordered discharge mechanism.

## 6. Natural Polymers for Drug Delivery

### 6.1. Chitosan Derivative

**Figure 7:**
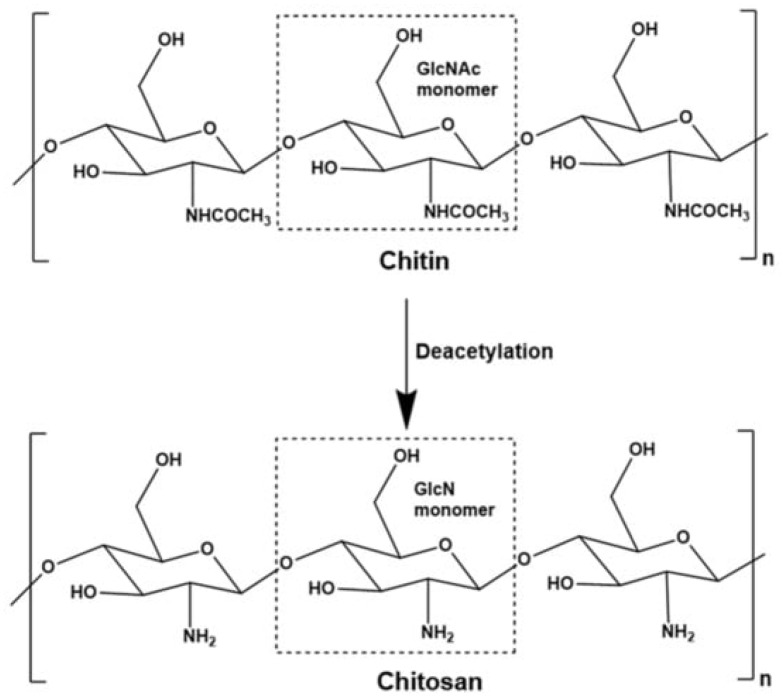
Mechanism of conversion of chitin to chitosan. (Reproduced from [93]).

A DDS regulates the distribution of pharmaceuticals inside a living organism in terms of both temporal and geographical parameters [90]. The objective of the DDS is to provide the appropriate quantity of medicine at the appropriate time and location in order to maximize bioavailability while simultaneously minimizing expenditures and adverse effects [91]. Within a DDS, the fields of medicine, engineering (including materials, mechanics, and electronics), and pharmaceuticals all interact with one another. The medication, drug carrier, and related delivery mechanisms are all a part of the research, as are any physical or chemical adjustments that were made to either the drug or the carrier [92]. Chitosan is derived from the chitin via deacetylation (Figure 7).

#### 6.1.1. Carrier for Deliveries

Chitosan and its derivatives are often dispersed by the use of micelles, gels, microspheres, and nanoparticles [94]. Microspheres have particle sizes that range from 1 to 500 μm, while nanoparticles have particle sizes that are less than 100 nm. Because of their diminutive size, nanoparticles are able to traverse a variety of biological barriers, paving the way for the targeted delivery of pharmaceuticals [95]. A micelle is a two-part structure that has exceptional stability, tissue permeability, and long-term drug release [96]. Micelles are characterized by their capacity to release drugs in a controlled manner. By producing a self-assembling micelle, amphiphilic chitosan has the potential to enhance the solubility of fat-soluble medications as well as their biological activity and the precision with which they may be administered [97]. A gel is a material that has undergone polymerization in three dimensions and has a crosslinked network structure. Gels have the ability to retain a significant amount of water. Gels that are flexible and malleable may boost chemical activity and dispersibility in biological fluids while also regulating stability, biodegradability, and biodegradability [98]. The mucoadhesion and permeability of gels are far higher than those of nanoparticles, and they also have the potential to transport very small molecules, all of which contribute to their use in a wide range of biological applications.

#### 6.1.2. Controlled Drug Delivery

Drug control and long-term release are two prospective study subjects [99]. Because certain medications have a short half-life and are swiftly absorbed, their plasma concentration drops. As a consequence, greater therapeutic dosages are necessary to maintain the plasma balance, increasing patient discomfort. Scientists are working hard to develop new pharmaceutical delivery methods that provide therapeutically acceptable medication concentrations [100]. Quick, constant, and long-lasting pharmacological release allows for rapid effectiveness while also offering long-term advantages [101]. A continuous, progressive release of chitosan-derivative nanoparticles is straightforward to accomplish, improving bioavailability and therapeutic effectiveness while decreasing negative effects [102]. While proteins are a suggested therapy for a variety of illnesses due to their accuracy and biocompatibility, protein treatments have certain disadvantages [103]. Proteins are quickly digested by enzymes, have minimal epithelial permeability in the intestine, and are poorly absorbed via the mouth. Protein medicines have limited utility due to these properties [104].

#### 6.1.3. Chitosan-Derivative Nanoparticles for Polypeptide Delivery

Strong hydrogen bonds and static electricity are required for the generation of peptide-loaded nanoparticles from chitosan-derivative nanoparticles interacting with peptides. Peptides that are not coated with nanoparticles are superior to their free counterparts in terms of temperature stability and the ability to be modified in vitro [105]. According to researchers, insulin-loaded, fatty acid-modified quaternary ammonium chitosan nanoparticles displayed a 98% encapsulation efficiency and loading capacity, which is superior to free insulin. This was shown by the ability of the nanoparticles to encapsulate insulin. When using insulin in a pill form, it is necessary to take into account the digestive and absorption capacities of the gastrointestinal tract [106]. Fucoidan (FD) is an amino acid that plays a role in the control of blood sugar. Trimethyl chitosan (TMC) and FD are responsible for the creation of nanoparticles that are then injected with insulin. TMC/FD nanoparticles are sensitive to pH, which allows them to block the breakdown of insulin while simultaneously boosting insulin cellular transport across the intestinal barrier [107]. In terms of insulin delivery, it has been shown that chitosan nanoparticles that have been treated with glycerol monocaprylate are comparable to TMC/FD nanoparticles [108].

#### 6.1.4. Chitosan-Derivative Nanoparticles for Gene Delivery

In terms of solubility, biodegradability, biocompatibility, nontoxicity, and transfection rate, nonviral vectors constructed of chitosan-derivative nanoparticles have performed better than chitosan nanoparticles [109]. Chitosan nanoparticles were also less toxic. It is essential for cancer treatments that genes have the capacity to change signaling networks and improve chemotherapy-induced tumour suppression [110]. Nucleic acids that are not packed tightly are unable to pass across cell membranes and are quickly nuclease-degraded [111]. It is possible for TMC to be modified in such a way as to prevent serum genes from being degraded by nucleases [112,113]. DOX and CA were covalently bonded to methoxy PEG-modified TMC (mPEG-TMC) to form mPEG-tetracyclododecene (TCD) nanoparticles, which are more effective against tumours than DOX and plasmid DNA on their own [114]. Studies conducted in vitro have shown that the presence of *O*-carboxymethyl chitosan nanoparticles inhibits the migration of tumour cells [115]. The cell transfection rates of poly(amino ester) and thiolated *O*-carboxymethyl chitosan composite nanoparticles with loaded genes were found to be greater than those of the former alone [116]. By altering chitosan derivatives, the target ligand may also be used to enhance targeted gene delivery. It has also been proven that the use of target ligands may increase tumour-specific delivery and cellular absorption and decrease adverse effects [117].

### 6.2. Alginate Derivatives

Alginate is a block copolymer composed of (1,4)-linked-*D*-mannuronate and *L*-guluronate monomers. It has been shown to be biocompatible and has the ability to produce hydrogels under conditions that are considered to be moderately physiologic. This has led to a great deal of interest in the application of alginate in biomedical research. Alginate is especially valuable in the distribution of medicines because its breakdown can be regulated, it is simple to chemically modify, and it has self-healing capabilities. Although previous chemical modifications of alginate for the administration of drugs have been documented, the objective of this study was to develop a method that maintains the inherent biocompatibility and non-toxic rapid crosslinking capabilities of alginate when combined with a divalent cation, such as calcium. This was accomplished by developing a technique that preserves these properties. There have been several applications for alginate-based scaffolds, including bone and tissue regeneration [118], wound healing [119], and drug delivery platforms [120,121,122,123]. Although alginate hydrogel microbeads may expand under physiological conditions, they are frequently solid and slow to break down [124], rendering them unsuitable for regulated therapeutic drug release in the gastrointestinal tract. Alginate hydrogel microbeads may expand under physiological conditions, and we have hypothesized that alginate microbeads containing basic functional groups will behave differently than those containing acidic functional groups when exposed to acidic or basic solutions [125,126,127,128]. As a consequence, modifying the release rate may be accomplished by elevating the alginate polymer capacity for swelling and/or its water-soluble properties at a certain pH. In research on hydrogel formation and features based on chemical and physical crosslinking polymers, the network structure and permeability of the material are often two of the primary factors [129]. In contrast, modifications to the vicinal hydroxyl group include the oxidation of alcohols to dialdehyde and the reductive amination of the oxidized alginate. Chemical adjustments to alginate include esterification [130], the Ugi reaction [131,132], and amidation [133]. The Ugi four-component condensation of an aldehyde, an amine, a carboxylic acid, and an isocyanide permits the production of α-aminoacyl amide derivatives in a short period of time. The Ugi reaction is exothermic and usually complete within minutes of adding the isocyanide. The products of the Ugi reaction may exhibit a broad range of substitution patterns and are peptidomimetics with potential medicinal uses. This reaction is thus very important for generating compound libraries for screening purposes. This was the first study to modify alginate by oxidizing the vicinal dialcohol backbone of the alginate to improve and better control the release rate of potential therapeutic substances in the gastrointestinal tract. Although a previous study modified alginate using three different bioactive peptide sequences (GRGDYP, GRGDSP, and KHIFSDDSSE) in combination with 8% periodate oxidized alginate, this was the first study to use the modified alginate [134].

### 6.3. Xanthan Gum (XG)

Xanthan gum (XG) is a polysaccharide with a high molecular weight that is produced via spontaneous fermentation. In 1961, the United States Department of Agriculture discovered the bacterium Xanthomonas campestris on cabbage plants. The bacteria were able to produce an extracellular polysaccharide that had favorable rheological characteristics. As a direct result of this finding, several significant advancements have been made in the production of polysaccharides. XG was the most widely used and widely accessible commercially produced microbial polysaccharide at the time [135]. In a wide range of different cosmetic and medicinal goods, XG is included as a component that serves the purpose of emulsifying and suspending [136].

### 6.4. Cellulose

One of the oldest plentiful biopolymers, cellulose is a potential material for nano-DDSs due to its cheap cost, high biodegradability, and remarkable biocompatibility [137]. In order to be effective, pharmaceutical delivery techniques need excipients that are not only bioavailable, but also biocompatible and biodegradable. Excipients based on cellulose and its derivatives have been the subject of a significant amount of study due to their green and natural qualities as well as their one-of-a-kind encapsulating and binding properties. Cellulose and its derivatives are widely used in controlled and long-term DDSs. Additionally, cellulose and its derivatives may change the solubility or gelling behaviour of drugs in a number of different applications, which results in a wide diversity of methods for regulating drug release patterns [138,139].

### 6.5. Cyclodextrin Derivative

PTX is an anticancer medication that has shown promise in treating a variety of different types of cancer. Because of its low water solubility and tendency to re-crystallize after being diluted, PTX is often manufactured with the assistance of co-solvents, such as Cremophor EL^®^, during the commercial production process. Amphiphilic cyclodextrins are chosen oligosaccharides for the administration of anticancer medications because they have the potential to spontaneously form nanoparticles and do not need a surfactant or co-solvent. Polycationic amphiphilic cyclodextrins have recently been produced as effective gene-delivery vehicles [140]. These cyclodextrins have been generated in the form of nanoplexes.

### 6.6. Hyaluronic Acid, Poly(Glycolic Acid), and Poly(Lactic Acid)

For the purpose of delivering drugs specifically to tumours, docetaxel (DCT) nanoparticles loaded with poly(*D*,*L*-lactide-*co*-glycolide) (PLGA) were employed to produce an HA acid–ceramide (HACE) nanostructure [141]. DCT-loaded PLGA nanoparticles were implanted into an HACE nanostructure to create nanoparticles with a limited size distribution and a negative zeta potential. This was accomplished using the DCT/PLGA/HACE formulation. PLA and PLGA have been used in the construction of vaccine, medicine, and gene delivery systems that are both safe and effective using manufacturing techniques that are well-described and easy to replicate [142,143]. With the help of these polymers, a wide range of nano- and micro-particulates may be produced. Table 1 summarized typical examples of natural and synthetic biomaterials.

## 7. A Revolutionary Nano-Biomaterial for Biomedical Purposes

Nanotechnology has shown promise in the medical field with regard to the optimization and production of one-of-a-kind nanoparticles. When it comes to biological applications, nanoparticles stand out as particularly useful in the detection, monitoring, and therapy of diseases or damage to tissue [144]. This capability is attributed to a wide range of physicochemical and biological features [145]. Polyester-based polymer nanoparticles have found use in the field of regenerative medicine, such as in imaging agents, systems for the administration of medicine, and multifunctional intelligent structures [146,147,148,149,150]. Nanoparticles that are connected to scaffolds, which are three-dimensional structures that support cells, have the potential to proliferate and heal damaged tissue. Poly(*L*-*co*-*D*,*L*-lactic acid-*co*-trimethylene carbonate) (PLDLA-*co*-TMC) is a high molecular mass polyester that is used in tissue engineering [151,152]. This material is biocompatible, biodegradable, and bioresorbable. When a polymer is combined with amphiphilic block copolymers, the efficiency of the initial material is preserved in terms of drug encapsulation and controlled release. Non-ionic block copolymers have several useful properties, including the ability to coat, stabilize, and self-assemble, as well as the ability to regulate the release of drugs. PEO-PPO-PEO is an abbreviation for poly(ethylene oxide) (PEO) and poly(propylene oxide) (PPO) [153,154,155]. When the physicochemical features of polymers are coupled, it is much easier to generate unique nanoparticles with attributes that are beneficial from a therapeutic standpoint. The characteristics of the final polymer nanoparticles are determined by the precursor polymer parameters as well as the preparation conditions, such as the amount of polymer and its molecular weight, the type of solvent, the addition sequence and phase concentration, the drip rate, and the amount of surfactant.

The solvent displacement technique, which is also known as nanoprecipitation, is one of the many methods that can be used to produce nanoparticles. This method has the potential to produce nanoparticles that are effective in encapsulating hydrophobic drugs at a low cost, with a simple production process, and with a low level of complexity. The degree of miscibility between organic (also known as the internal phase) and aqueous (also known as the external phase) solvents is what governs the nucleation, development, and aggregation of nanoparticles [156]. The Marangoni [157,158] and Ouzo effects are two hypothesized components of the molecular interfacial environment that are responsible for the generation of solutions. The Marangoni effect encourages the processing of nanoparticles by increasing flow, diffusion, and surface tension fluctuations at the interface between the solvent and non-solvent (water) [159]. Nanoparticle processing occurs when metastable liquid dispersions spontaneously emulsify through liquid–liquid nucleation. When processing polymer nanoparticles, it is essential to have an understanding of the effect of molecular interface variables and physicochemical parameters on the process [160]. Quality is achieved by design and process optimization, which includes the design of trials for product development backed by the industry. As a direct result of this, producing perfect nanoparticles and developing a strategy that can be replicated on a large scale will be simple tasks. Statistical methods for analysing nanoparticle formation processes, such as the factorial 23 and Box–Behnken designs [161], have been shown to be useful in evaluating the effects of independent variables on responses and forecasting changes in NP physical–chemical properties, such as hydrodynamic diameter, polydispersity index, and zeta potential [162].

## 8. Consideration of General Mechanisms

### 8.1. Tissue-Targeting Design, Surface Functionalization, and Controlled Release

Passive (increased accumulation owing to passive physiological variables) and active (application of ligands to a specific target) diffusion are the two methods that may be used for concentrating medications to a particular area of interest [163]. The surface modification of drug carriers with bioactive compounds that interact with cell receptors and that are adsorbed, coated, conjugated, or connected to them demonstrates a preference for a certain cell or tissue type, which may increase medicine absorption. The surface modification of drug carriers with bioactive compounds can be done in a number of different ways. It is possible to apply modified coatings (for instance, ones that include albumin and chitosan) in order to limit the enzymatic breakdown that occurs in the gastrointestinal tract and plasma [164].

Both non-antibody ligands, such as lectins (carbohydrates that are adapted for cell surfaces) and monoclonal antibodies, have been put through their paces in this type of research [165]. Recent research has shown that small molecules or peptides that function as agonists/subtracts or antagonist inhibitors for overexpressed receptors on the cell surface of particular organs have shown potential for concentrated drug delivery [166,167]. There are a number of considerations that need to be made, one of which is the use of targeting ligands, which have the potential to boost distribution to secondary target sites in tissues other than those that are initially targeted [168]. On the other hand, ligands that are not antibodies have the drawback of not being selective in their binding [169]. Immunoconjugates pose concerns about immunogenicity and reticuloendothelial system (RES) retention [170]. Coating the carrier surface is an option for modifying the lipophilicity and hydrophilicity profile, lowering the rate at which immune cells are absorbed, and enhancing cell identification, e.g., the synergy between the distribution and signaling of antibodies. Within minutes after receiving an IV injection, nanoparticles were eliminated from the plasma as a result of opsonization, which was followed by phagocytosis carried out by RES cells. It is possible that surface ligands might assist in reducing opsonization. PEG is a hydrophilic polymer that has been shown to increase plasma protein resistance while simultaneously reducing serum aggregation brought on by ions and proteins [171,172]. Reduced immune responses may be achieved by preventing phagocytes from opsonizing and detecting the invading pathogen. PEG may also inhibit enzymes from accessing dendrimer scaffolds, which results in a reduction in the rate at which dendrimer is broken down [173]. PEG-coated in vivo nanoparticles and liposomes extend the amount of time it takes for an individual’s blood to circulate, from minutes to hours [174,175]. The surface density of PEG, the length of its chain, and its capacity to inhibit hepatic absorption all contribute to the efficiency of the compound. On the other hand, PEG carriers are designed to infiltrate cells, and the presence of PEG may prevent the carrier from interacting negatively with cells in some circumstances. PEG carriers are meant to enter cells. PEGylated nanocarrier systems have also been connected to the accelerated blood clearance (ABC) phenomenon, which causes increased accumulation in the liver and spleen after repeated injections [176,177]. This accumulation occurs because PEGylated nanocarrier systems are lipid-coated. It has been proven that the ABC phenomenon activates an immune response known as the ABC response. This reaction causes greater accumulation in the liver and spleen after repeated injections, which is the phenomenon known as ABC.

### 8.2. Simultaneously Encapsulated Drugs for Combined Therapy

Simultaneously encapsulated drugs are drug delivery devices that can simultaneously administer many medications to deliver efficient chemotherapeutic drugs while suppressing the P-glycoprotein (P-gP) efflux pathway. P-gP is considered an obstacle to the successful pharmacotherapy of cancers because this protein pumps the drugs out of the cells. Consequently, P-gP overexpression is one of the main mechanisms behind decreased intracellular drug accumulation and the development of multidrug resistance in human multidrug-resistant cancers. PLGA nanoparticles were loaded with vincristine sulfate and verapamil hydrochloride at the same time and, by overcoming tumour insensitivity, boosted the therapeutic index. As a result, the same approach was used to administer DOX and cyclosporine-A [178,179]. According to current research, PLGA-PEG interacts with P-gP [180] to potentially improve system efficiency. When constructing these systems, the features of the pharmaceuticals to be encapsulated must be considered. Hydrophobic drugs, for example, can be encased in hydrophobic polymers [181]. This limitation may be solved by utilizing novel polymers, such as PLA-PEG-PLA or PCL-PEG [182], which have been successfully employed to encapsulate retinoic acid and calf-thymus DNA [183]. Another way to improve therapy is to use two different drug release rates (such as in cancer treatment). A PTX and a C6-ceramide were encapsulated in a bespoke mixture of poly(lactic-*co*-glycolic acid) and poly(*β*-amino ester) (PLGA/P*β*AE) [184] to effectively overcome cancer treatment resistance mechanisms.

### 8.3. Carrier Distribution

RES, which are found largely in the liver and spleen, are the most important barriers to carrier systems [185]. This is because of their inclination to internalize and withdraw themselves from systemic circulation. The concentration of PLGA NPs was greatest in the liver (40%), followed by the kidney (26%), the heart (12%), and the brain (13%), with just a small proportion present in the plasma [186]. PLGA/PbAE yielded findings that were comparable [187]. The method of administration has no effect on the distribution pattern since lymphatic clearance occurs after intraperitoneal (IP) injection and applies to all kinds of charged particles [188]. Additionally, carrier lipophilicity affects cell absorption by causing more hydrophilic particles to be expelled more rapidly [189]. This has an effect on how well the cell can take up the carrier. The nanoparticle charge surface and route of administration were studied using 10 nm gold nanoparticles that had been functionalized with a variety of groups, aiming for distinct zeta potentials (neutral, negative, positive, and zwitterionic). IV and IP administrations were utilized. Following IV administration, the peak plasma concentration of positively charged particles was ten times lower than before, and both negatively and positively charged particles were eliminated from the body more quickly [190,191]. Following IP injection, only a minute amount of both positively and negatively charged particles could be identified in the sample [192]. According to these data, the circulation was enhanced by neutral and zwitterionic nanoparticles. The significant discrepancies in bioavailability may be explained by the opsonization of nanoparticles with antibodies for detection by local macrophages [193] as well as by a similar discovery in dendrimers [194]. Nanocarriers that can be manipulated have the potential to deliver medications to specific organs and tissues. Dendrimer branch size may be adjusted to affect dendrimer dispersion and removal. As a consequence of this, dendrimers could avoid renal clearance using a cut-off that is between 40 and 60 kDa, just like the G7 [195,196]. G1 to G5 dendrimers are quickly evacuated to the kidneys and bladder, whereas G3 to G7 dendrimers are regularly recognized in circulation [197]. G8 dendrimers may be found in the lymph nodes, and G9 dendrimers are identified in the liver. As mentioned earlier, PEG plays a role in determining how evenly the carrier is distributed throughout the body. As the molecular weight of the PEGylated dendrimers rises, uptake from the injection site into the lymph becomes a considerable contribution to the total absorption profile. This indicates innovative drug delivery options as well as increased lymphatic system imaging agents [198,199].

## 9. Drug Delivery Using Mucoadhesive Hydrogels

### 9.1. Mucoadhesive Biomaterials

In the early 1980s, Nagai [200] came up with a new way to treat aphthae at the local level with an adhesive tablet. This sparked a lot of interest in developing mucoadhesive DDSs. Nagai also discovered that using a mucoadhesive polymer enhanced the absorption of peptides when administered nasally [201]. There has been a lot of pioneering work in this field, such as the development of mucoadhesive creams based on poly(acrylic acid) (PAAc) [202] and poly(methyl methacrylate) [203]. The majority of early research on mucoadhesion was done with traditional polymers in the form of tablets [204], powders [205,206], or films [207]. The promising results observed during the creation of these early mucoadhesive formulations suggested that mucoadhesion should be investigated further. The advantages of mucoadhesive devices over traditional drug delivery methods should certainly be exploited.

Biomaterials, such as synthetic and natural polymers, have been used in the creation of innovative mucoadhesive medicinal devices to date. PAAc and cellulose derivatives make up the majority of currently used synthetic mucoadhesive polymers. Polymers that are seminatural, such as chitosan, gellan carrageenan, and pectin, are also included in the list of seminatural mucoadhesive polymers. There are also other synthetic mucoadhesive polymers, such as poly(*N*-vinyl pyrrolidone) (PNVP) and PVA [208]. The most widely used polymers for the preparation of hydrogels are listed in Table 2.

At the molecular level, the chemical structures of already-existing mucoadhesive polymers could be improved. Kali et al. [209] suggested the incorporation of thiol groups into polymers in order to enhance mucoadhesion. As a result of the formation of disulfide bonds with cysteine-rich regions in mucins, polycarbophil-cysteine conjugates exhibit a great deal of mucoadhesion. The discovery of lectins was yet another groundbreaking achievement. Proteins known as lectins possess a great degree of selectivity in the carbohydrates to which they bind. The second generation of mucoadhesive uses lectin-conjugated polymers to attach to receptors on epithelial cell surfaces. Because of their capacity to bind to cellular structures, these polymers are often referred to as cytoadhesives. In spite of the fact that this polymer property differentiates it from mucoadhesive polymers, it should be noted that in order for the polymer to reach the epithelial cell membrane, it must first diffuse through the whole mucus layer. As mentioned earlier, one of the functions of this mucus layer is to serve as a protective barrier for the cells that lie underneath it. In some parts of the body, the mucus coat could be up to 400 μm thick. In order for cytoadhesive polymers to be able to reach the epithelial surface, they must first be able to selectively adhere to the cell membrane and then spread through the mucus layer.

The theory of diffusion is now being used in an effort to provide an explanation for some of the additional molecular changes that are utilized in mucoadhesive polymers. In 1963 [210], Voyutskii proposed the diffusion hypothesis as an explanation for the adherence of rubbery polymers. According to this idea, a chemical potential gradient leads the polymer chains to extend beyond the initial barrier whenever two rubbery polymers come near enough to one another. A polymer–mucus system is formed when polymer chains form and interweave with mucin glycoproteins. This results in the formation of a polymer–mucus system. In the context of mucoadhesion, Peppas and colleagues [211] suggested the interdiffusion hypothesis. They reasoned that increasing chain interpenetration would lead to an improvement in mucoadhesion. Minghao et al. [212] used ATR/FTIR spectroscopy to investigate the mucin interpenetration that occurred at the PAAc/mucin interface. According to their results, the amount of mucin present in the polymer made from PAAc increased as more time passed. Nicholas and Mikos et al. [213,214] supported the use of sticky promoters in order to improve chain interpenetration and, as a result, polymer mucoadhesion. Their reasoning was based on the notion of chain interpenetration. The injection or grafting of adhesion promoters may be done into the surface of the matrix.

**Figure 8:**
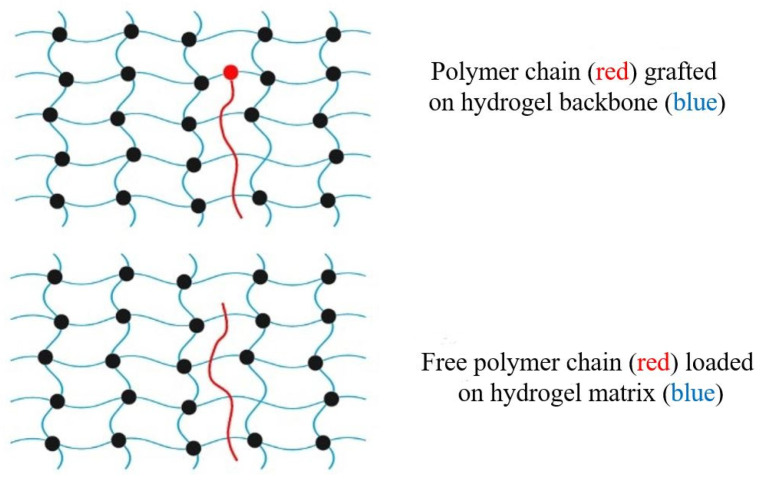
Schematic illustration of polymer chains used as adhesion promoters in hydrogel matrixes.

Morello et al. [215] used a theoretical approach in their research on the interpenetration of free chains in mucoadhesion. They found that the length of the chain as well as the percentage of gel volume impacted the mobility of the distributed chains. Figure 8 illustrates how free polymer chains may be grafted onto the backbone of a hydrogel in order to assist in the ability of the hydrogel to adhere to other surfaces. When free adhesion promoters are brought into direct contact with mucus in a mucoadherent device, this causes the chains to scatter, which results in a concentration gradient being created in the device. Effective interactions, such as hydrogen bonding and physical entanglements, manifest themselves at the point of contact [216]. According to Haimhoffer et al. [217] the mucoadhesive properties of the polymer were improved by the incorporation of free PEG chains into crosslinked PHEMA particles. These chains served as adhesion promoters. It was hypothesized that free PEG chains moving over the interface were the cause of the problem.

The black-filled circles in Figure 8 indicate cross linking of the polymers. Cross-linking changes a liquid polymer into a “solid” or “gel” by restricting movement. There are two types of crosslinking: physical and chemical. Physical crosslinking may not be permanent, but chemical or permanent hydrogels are formed by the covalent crosslinking of polymers. The hydrogel backbone may be grafted with polymer chains, or the network can be inserted freely. Sahlin and Peppas [218] used near-field FTIR microscopy to investigate the diffusion of free PEG chains through a PAAc hydrogel. The ability of linear PEG chains to improve mucoadhesion via interpenetration in hydrogels was first established in 1997 [219]. Tethered polymer chains may also be used to make adhesion promoters. For diffusion and penetration, the tether is chemically linked to one end of the hydrogel surface and left unattached on its opposite end. While in close contact with mucus, grafted chains are able to expand across the interface because of a concentration gradient. Covalent bonds between the backbone hydrogel structure and the adhesion promoter chains prevent them from being lost. The polymer chains of the adhesion promoter may be able to cross mucus and connect tissue to a mucoadhesive device [220,221]. Further uses for surface-anchored polymers have been discussed. For example, the single-chain mean field (SCMF) theory was used by Huang et al. [222] to investigate tethered polymer gel–gel adhesion. Theoretical developments assisted in the creation of novel tethered polymer chain biomaterials. Analysis of interactions between PEG chains and mucin glycoproteins was performed using a surface-force instrument. PEG-tethered structures might also be used to develop new mucoadhesive drug delivery systems in the future, according to their results.

### 9.2. Mucoadhesive Medication Delivery with Hydrogels

Water-soluble and water-insoluble systems coexist in mucoadhesive polymers. Mucoadhesive polymers that are water soluble are typically linear or branched polymeric molecules. The rate at which they dissolve determines how long they stay in the water. Swellable networks having a crosslinked chemical structure are known as water-insoluble polymer networks. The residence period of water-insoluble mucoadhesive biomaterials is determined by mucus turnover or cell desquamation. When exposed to water or physiological fluids, hydrogels form three-dimensional polymer networks [223,224]. Hydrophilic homopolymers or copolymers are used to make these networks. The crosslinks in the chemical structure keep them from dissolving and give them a distinct physical integrity. Hydrogels have been widely employed in medical and pharmaceutical fields because they better imitate genuine tissue than any other synthetic biomaterial. Because of their high-water content and flexible structure, hydrogels are biocompatible [225,226]. Contact lenses, biosensor membranes, prosthetic skin, artificial heart linings, and drug-delivery devices have all been made using hydrogels [227,228,229,230,231,232]. The network architecture of a hydrogel determines the characteristics of a drug delivery system. Three essential parameters may be used to understand the network structure of hydrogels:

✓ The swelling ratio, including the mass swelling ratio and the volume swelling ratio;
✓ The polymer volume fraction in the swollen state;
✓ The number-average molecular weight between cross-links (*M*_c_);
✓ The network mesh size.

**Figure 9:**
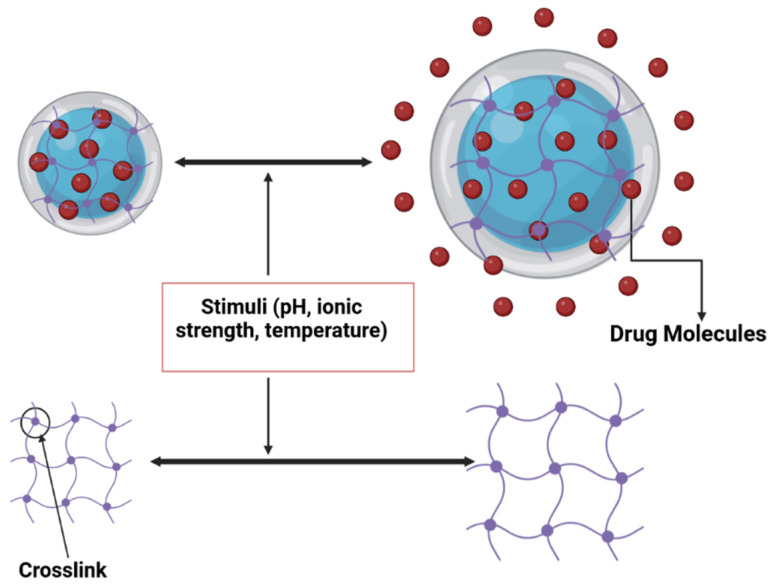
Physiologically sensitive hydrogel: certain external events cause the drug to swell and release.

The swollen polymer volume fraction is used to determine the quantity of fluid that the hydrogel can absorb and hold. Molecular weight differences between crosslinks dictate the degree of crosslinking. Since polymerization is a stochastic process, only average *M*_c_ values may be derived. The distance between crosslinks or connections is determined by the mesh size, which regulates the amount of available drug diffusion space between macromolecular chains. Hydrogels may be either neutral or ionic depending on the kind of charge in their pendant groups. The swelling of hydrogels may also be caused by the surrounding environment. Recent years have seen a surge in interest in hydrogels that are responsive to body chemistry [233,234]. Many factors, including temperature, electromagnetic radiation, and pH, may affect the swell ability of hydrogels (Figure 9).

pH-sensitive hydrogels, which feature swelling behaviour and a three-dimensional architecture that are affected by the external environmental pH, employ acidic or basic pendant groups. Some chemical groups ionize as the pH and ionic strength of the environment change, causing structural changes in hydrogels. Because medicine may be delivered to particular parts of the body while simultaneously being shielded from potentially harmful biological circumstances, these qualities make pH-sensitive hydrogels ideal for use in the development of DDSs. As a medication delivery and protein control strategy, complexation hydrogels have also been previously studied.

At present, bacterial cellulose is gaining more interest to researchers due to it having a wide variety of current and potential future applications. Due to its many unique properties, it has been used in the food industry, the medical field, commercial and industrial products, and other technical areas. In the medical industry, bacterial cellulose-based hydrogels are attractive materials for wound dressing applications due to their hydrophilic properties, purity, ability to maintain appropriate moisture balance, and flexibility in conforming to any contour of the wound, forming a tight barrier between the wound and the environment, thus preventing bacterial infections. It also found its place in tissue engineering applications because of its biocompatibility, non-toxic effects, porous structure, and good mechanical strength [235,236].

The hydrogel is distinguished by the joining of chemical groups from several polymer chains. Hydrogen bonding [237,238,239,240,241,242] is one of the interactions that results in these chemical links between macromolecular chains. A wide range of monomers have been used to make therapeutic hydrogel biomaterials. Hydrogel topologies may be created by combining different monomers. Table 2 lists some of the monomers often utilized to make hydrogels for medical and pharmaceutical applications. Polymer chains are fused together to form the hydrogel [242]. Hydrogen bonding is a method through which macromolecular chains form chemical bonds. To create hydrogel biomaterials for therapeutic reasons, many monomers have been utilized. Hydrogels for medical and pharmaceutical applications may be made from any of the monomers mentioned in Table 2. It is incredible that so many different kinds of hydrogel structures can made, each with unique physical and chemical properties. To achieve precise drug delivery goals, the molecular design of customized biomaterials has exceeded the strategy of converting “off the shelf” polymeric materials for consumer usage to therapeutic use. As shown in Table 3, there are a number of hydrogel polymers that might be used in the administration of medicines under strict supervision. There are hydrogels that are able to cling and stick to mucosal surfaces, making the medication delivery device persist longer. The mucoadhesive qualities of a biomaterial are determined by the chemical composition and topology of the hydrogel, as previously stated.

### 9.3. Mechanisms of Drug Release from Mucoadhesive Hydrogels

To deliver therapeutic pharmaceuticals, a mucoadhesive hydrogel may provide suitable drug release rates and locate and hold the pharmaceutical device in a particular location of the body for an extended period of time. In comparison to typical medicinal devices, mucoadhesive hydrogels provide a number of advantages. Understanding how a medication releases over time is crucial in the science of drug distribution. To construct novel pharmacological formulations and evaluate experimental data, mathematical models are needed [243]. The majority of theoretical models used to analyse pharmaceutical transport rely on simple diffusion equations. Controlled drug release systems are available in a wide range of forms since medication may be administered in a number of ways [244].

- DDSs that are controlled through diffusion;
- Chemically controlled DDSs;
- Swelling-controlled DDSs.

Each of these systems will be addressed in this section. A quick review of diffusion fundamentals is also included, as diffusion plays a significant part in all of the systems.

#### 9.3.1. Diffusion Fundamentals

Drug molecules must pass through the polymer bulk to be released from a mucoadhesive hydrogel. Using mass transportation concepts, we can explain the occurrence of diffusion phenomena. Drug molecules are transferred from a hydrogel matrix to an external environment using Fick’s equations of diffusion. The first and second of Fick’s diffusion laws are represented in Equations (1) and (2), respectively. The Fickian rules are shown in a flat manner:

(1) $$J=-D\frac{dC}{dx}$$

(2) $$\frac{\partial C}{\partial t}=D\frac{\partial^{2}C}{\partial x^{2}}$$

where *c* and *J* represent concentration and mass flow, respectively, and *x* and *t* represent the independent variables of location and time, respectively. The diffusion coefficient (*D*) is the unit of measurement. Equation (1) of Fick’s first law of diffusion is used when the steady state has been achieved or when the drug concentration within the diffusion volume does not fluctuate over time. This may be shown by using a zero-order drug release model. Equation (2) of Fick’s second equation of diffusion is used to describe diffusion when a steady state has not been achieved or the concentration within the diffusion volume fluctuates with time. In this equation, ∂*C* is the change in *C*, ∂*t* is the change in *t*, and ∂*x* is the change in *x*.

#### 9.3.2. Drug Delivery Systems with Diffusion Control

There are two types of diffusion-controlled systems: monolithic and reservoir. In monolithic devices, the medication is tightly attached to the polymer, which possesses rate-controlling properties. When the polymer has been dissolved or distributed, two monolithic systems may be formed. No matter how long a medicine stays in the system, zero-order kinetics cannot be applied to therapeutic compounds. Reservoir devices have a polymeric membrane that controls the pace at which the medication is delivered. Medications are transported from the polymer core to the polymer membrane by dissolution at one interface and diffusion caused by a change in thermodynamic activity. Using Fick’s first law, Equation (1) depicts the movement of drugs. If the thermodynamic activity of the drug in the reservoir is constant and infinite sink conditions are maintained, the release rate of the drug will be constant and predictable. To achieve zero-order kinetics, the medication will be administered at a constant rate. Fick’s first law may be recast to predict drug release rates from planar devices, as shown in following Equation:

(3) $$\frac{dM_{t}}{dt}=\frac{ADK∆C}{t}$$

where d*M*_t_/d*t* represents the rate of drug release (mass/time), *A* is the membrane surface area, *D* is the diffusion coefficient, *K* is the partition coefficient of the drug in the membrane, and Δ*C* is the concentration gradient. Similarly, the restated Fick’s first law may be used to calculate drug release rates from cylindrical delivery devices:

(4) $$\frac{dM_{t}}{dt}=\frac{2\pi hDK∆C}{ln(r_{0}-r_{1})}$$

where *r*_0_ is the interior radius, *r*_1_ is the exterior radius, and *h* is the height. It is possible to analyse drug release rates from spherical delivery systems using a rederived version of Fick’s first law:

(5) $$\frac{dM_{t}}{dt}=4DKC\frac{r_{0}r_{1}}{\left(r_{0}-r_{1}\right)}$$

According to these equations, the shape of the delivery system may be used to control the release of pharmaceuticals. If you know the polymer structure and the partition coefficient of the system, you may determine how much medicine will be released through the membrane as well as the thickness of the membrane.

#### 9.3.3. Drug Delivery Systems with Swelling Control

**Figure 10:**
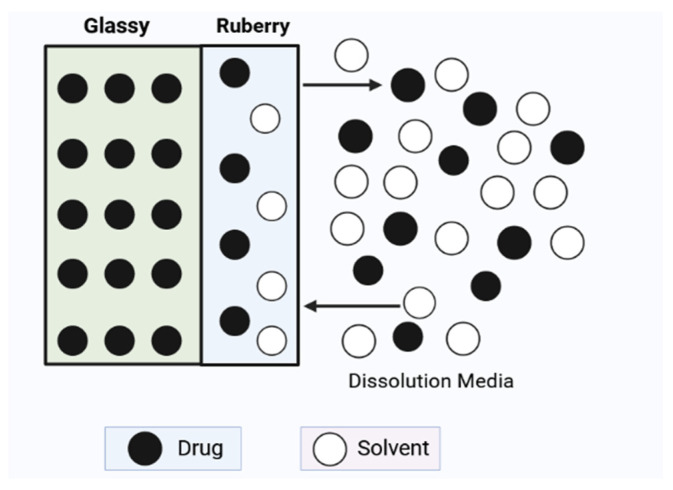
Depiction of a drug delivery system whose swelling is controlled. The polymer matrix swells and the rubbery phase develops when water comes into contact with the glassy hydrogel. Drug molecules can scatter out after passing through the rubbery phase.

When water enters a hydrophilic polymer matrix, it causes the device to swell, which in turn controls the amount of medication released. A glassy polymer matrix first distributes the drug in an even manner. The medication is held in place by glassy polymers that are almost impenetrable. Drugs cannot disperse because of the glassy nature of the polymer. A rubbery phase is formed when the polymer matrix expands in the presence of water or biological fluids. Inner glass and exterior rubber phases are both visible. Drug compounds may be more difficult to detect if they go through the rubbery phase (Figure 10). In order to control the quantity of medication discharged, the pace and location of the rubbery front need to be adjusted. Swelling-controlled devices may disperse medication through diffusion and polymer chain relaxations at the glassy–rubbery interface [245]. Ritger et al. [246] provided a simple equation for evaluating diffusion and macromolecular relaxation:

(6) $$\frac{M_{t}}{M_{\infty }}=kt^{n}$$

where *M*_t_/*M*_∞_ is the fraction of drugs released, *M*_t_ is the amount of drug released over time *t*, *M*_∞_ is the amount of drug at the equilibrium state, *k* is the rate constant, and *n* is the diffusional exponent characteristic of the release mechanism. This exponential equation [243,246] for drug release from polymeric matrixes was developed after both Fickian and non-Fickian diffusional behaviour were researched and the implications on exponent n of these two types of diffusion were investigated. Dispersion coefficient values were shown to be connected with drug delivery systems that were either flat, cylindrical, or spherical. Table 4 summarizes these results.

## 10. Natural and Synthetic Biomaterials to Deliver Extracellular Vesicles (EVs)

**Figure 11:**
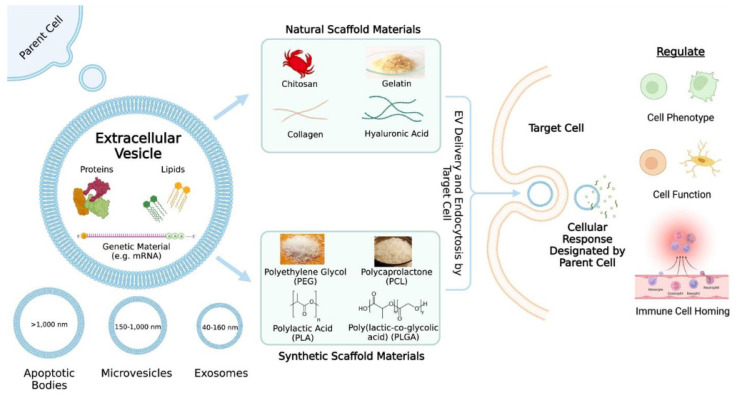
An overview of extracellular vesicle (EV) delivery via scaffolds. The contents and categories of the extracellular vesicles as well as what the extracellular vesicles may regulate are described. Common natural and synthetic biomaterials for scaffold fabrication are highlighted (Reproduced form [264]).

Extracellular vesicles (EVs) are nanosized membranous structures derived from cells and can be classified into subclasses, such as exosomes, microvesicles, and apoptotic bodies. Exosomes, formed through the inward budding of endosomes within multivesicular bodies (MVBs), are released when MVBs fuse with the plasma membrane (Figure 11). Researchers often categorize EV subclasses based on physical characteristics, biochemical composition, conditions, or cell of origin due to challenges in capturing live images of EV release [247,248]. EVs play various roles in physiological and pathological processes, including suppressing inflammation, modulating cellular function, regenerating tissue injuries, and influencing the immune system. The specific functions of EVs depend on their origin, and when taken up by target cells, they release contents such as proteins, lipids, and genetic material, leading to changes in gene expression. The passage emphasizes the importance of studying reliable markers for EV subtypes to establish a consensus on nomenclature. Additionally, it highlights the potential benefits of using EVs in bioengineering, as their contents allow for the regulation of phenotype, function, and immune cell homing. Specific EV cargoes related to positive therapeutic outcomes are explored in subsequent sections [249].

There are two main types of biopolymers that are utilized in bioengineering: natural and synthetic. Natural biomacromolecules, such as silk fibroin, collagen, gelatin, chitosan, and hyaluronic acid, are explored, along with widely studied synthetic biopolymers, such as PEG, PCL, PLGA, and PLLA (Figure 11). Each type has its benefits and challenges; natural biomaterials may vary and have potential issues with mechanical stability, while synthetic biomaterials lack native tissue structure and may pose toxicity risks [250].

Here, we will highlight some natural and synthetic biopolymers for therapeutic EV delivery. Sodium alginate is a linear polysaccharide derived from brown seaweed, specifically a derivative of alginic acid composed of *α*-1-guluornic and 1,4-linked-*β*-D-mannuronic monomers. Alginate-based hydrogels loaded with EVs have been investigated for various therapeutic purposes, such as healing diabetic wounds [251], regenerating peripheral nerves [252], and addressing myocardial infarction [253]. Silk fibroin (SF) is a hydrophobic protein derived from the Bombyx mori silkworm known for its self-assembling properties that create strong and resilient materials. Cunnane and colleagues [254] investigated the impact of extracellular vesicles derived from human adipose-derived mesenchymal stem cells on vascular cells in an in vitro setting. Their findings revealed that the use of these EVs led to a dose-dependent enhancement in both the proliferation and migration of smooth muscle cells and endothelial cells. Chitosan is a cationic polysaccharide derived from chitin and composed of di-glucose amine and *N*-acetyl glucose amine groups. Scaffolds made from chitosan have been employed for the administration of EVs to enhance the reparation of bone defects, address corneal diseases, facilitate the healing of skin wounds, and treat injuries to articular cartilage. Wu and colleagues [255] specifically created chitosan-based thermosensitive hydrogels loaded with small EVs derived from bone mesenchymal stem cells to expedite the processes of osteogenesis and angiogenesis. Collagen, the predominant protein in mammals, is formed with three interwoven α-chains. Scaffolds made from collagen have been applied for therapeutic objectives, such as bone and endometrium regeneration. Xin and colleagues [256] developed a collagen scaffold incorporating exosomes derived from umbilical cord-derived mesenchymal stem cells to facilitate endometrial regeneration in a rat model of endometrial damage. HA is a linear polysaccharide composed of repeating units of *D*-glucuronic acid and *N*-acetyl-*D*-glucosamine. Scaffolds based on hyaluronic acid have been employed for delivering EVs as a treatment for tendon repair and injuries to cartilage in osteoarthritis. K. Song and colleagues [257] isolated exosomes from tendon-derived stem cells, loaded an HA scaffold with these exosomes, and investigated the therapeutic effects of this system for tendon repair. Gelatin, a commonly employed natural biopolymer in the fields of regenerative medicine and tissue engineering, has found application in scaffolds for bone regeneration. In their study, Man and colleagues [258] enhanced the osteoinductive potency of osteoblast-derived EVs through epigenetic modification using the histone deacetylase inhibitor Trichostatin A. PEG is an FDA-approved polymer known for its hydrophilic and flexible characteristics and deemed safe for biomedical applications. PEG hydrogels have been employed to deliver exosomes, aiding in the healing of cutaneous wounds [259]. PCL, an aliphatic polyester characterized by its linear nature, hydrophobic nature, high mechanical strength, and biocompatibility, is also biodegradable. Wei and colleagues explored the potential of heparin-functionalized vascular PCL grafts to improve their anti-thrombogenic properties. The researchers manufactured tubular PCL grafts through electrospinning, introduced heparin modifications to the grafts, and incorporated small extracellular vesicles derived from mesenchymal stem cells by immersing the scaffolds in an EV solution [260]. PLGA is a copolymer that shares similarities with PCL, being a biocompatible, biodegradable, and flexible biopolymer. Research has explored the use of PLGA scaffolds loaded with EVs to enhance the treatment of bone defects and chronic kidney disease. Ko and colleagues [261] specifically developed a PLGA-based scaffold for the delivery of EVs derived from stem cells, aiming to promote kidney regeneration. PLLA undergoes degradation through nonenzymatic hydrolysis, and its resulting by-products are eliminated through regular cell metabolism. Swanson and colleagues devised a biodegradable delivery platform based on PLLA to regulate the release of exosomes from microspheres, aiming to enhance craniofacial bone healing. In their approach, they employed PLGA and PEG triblock copolymer microspheres to encapsulate and control the timed release of exosomes derived from human dental pulp stem cells [262]. PLA is both biocompatible and biodegradable through hydrolysis and enzymatic activity, and it exhibits high hydrophobicity. In their work, Gandolfi and colleagues sought to create a mineral-doped, PLA-based scaffold that could be functionalized with EVs to enhance the osteogenic commitment of human adipose-derived mesenchymal stem cells. The researchers observed that mineral-doped PLA scaffolds effectively adsorbed red-labeled exosomes derived from human adipose mesenchymal stem cells [263].

## 11. Pharmaceutical Applications

Pharmaceuticals based on nanotechnology have grown in popularity and have had a significant impact on the pharmaceutical industry. Compared to other industries, nanoparticle technology is a significant amount of the nanotech pharma business. The electro-spraying method addresses scalability, reproducibility, efficient encapsulation, and other potential nanoparticle production needs. Pharmaceuticals electro-sprayed with and without polymer-carriers have been employed in a wide range of applications. Drug release quality is improved by using biodegradable polymer-carriers to delay the release of encapsulated medications. In pharmaceutical applications, electro-spraying is a popular approach for generating nanoparticles [265]. Polymers have played an integral role in the advancement of drug delivery technology by providing a controlled release of therapeutic agents in constant doses over long periods, cyclic dosage, and tuneable release of both hydrophilic and hydrophobic drugs. The types of polymers used in the pharmaceutical industry must meet the same safety requirements, including sterilizability, biocompatibility, processability, fluid compatibility, and an optimum balance of mechanical properties tailored for the given application.

### 11.1. Brain Delivery

To sustain homeostasis and other vital tasks, the brain is a fragile neuronal organ system that requires a steady supply of fuels, gases, and nutrients. The blood–brain barrier (BBB), which is a type of vasculature in the central nervous system that acts as a physical barrier, is responsible for a number of problems. The BBB makes it more difficult for therapeutic drugs to reach the brain and spinal cord [266]. Antibiotics, anticancer drugs, and neuropeptides are among the types of pharmaceuticals that cannot get past endothelial capillaries and into the brain. For central nervous system (CNS)-related disease therapy, several pharmacological delivery systems and procedures have been devised. However, the majority of these procedures have been shown to be intrusive and lack target selectivity. Regardless, all prior medication delivery systems have been developed by trial and error. These are nearly always used to deliver a small number of medications with good structure–activity correlations, drug-receptor linkages, and structure-transport ties [267]. When it comes to allowing medications to pass through by diffusion or active transport, the BBB is nonselective, posing significant challenges for CNS drug development. The brain, on the other hand, quickly absorbs glucose and fat/lipid soluble medications. Due to their fat-insoluble nature, certain drugs, contrary to popular belief, are difficult to transfer into the brain. Pharmacological availability at potentially deadly doses is reduced because the brain capillary endothelium only distributes a small amount of medication [268]. CDs are employed in pharmaceutical applications for a variety of reasons, including improving medication bioavailability. Current CD-based therapies have been explored and potential future uses provided [269]. Additionally, various carrier materials are continually being developed to overcome the limits of therapeutic medications [270]. Cyclodextrins have been identified as attractive candidates due to their capacity to impact the physical, chemical, and biological features of guest molecules through the formation of inclusion complexes [271].

### 11.2. Mucosal Drug Delivery

The mucus layer works as a barrier when drugs are administered to mucosal surfaces despite the fact that it is typically ignored. The mucus gel layer that surrounds the mucosal epithelia forms a barrier [272] and has been shown to be an important component of the mucus layer owing to its water content of roughly 83% [273]. The apical location of a cell is connected to its mucus layer, which acts as a barrier for the cells below [274]. Several investigations have shown that chitosan has excellent mucoadhesive properties, which have been researched extensively. A variety of therapeutic chitosan derivatives have also been tested for their ability to adhere to the mucous membrane, with the purpose of identifying how structural modifications impact mucoadhesion [275,276]. Several processes will be discussed in this review, including ionic interactions with mucin chains and the hydration state of chitosan. Three-dimensional printing has been used to build single- and multi-layered medicine delivery systems in addition to its most common usage in oral delivery. Additionally, oral mucosa-based administration is possible through buccal and sublingual routes. Squamous cells lining the buccal cavity have a surface area of 50 cm^2^ and an average thickness of 500–800 μm. Sublingual epithelium on the bottom of the mouth may potentially absorb drugs. The lower thickness of non-keratinized sublingual epithelium (100–200 μm) and its substantial vascularization frequently result in better drug penetration and early onset when compared to the buccal route.

### 11.3. Pulmonary Drug Delivery

Pulmonary medicine distribution is a prominent topic in research because the lungs may absorb medications for both local and systemic administration. Respiratory epithelial cells are in charge of both the production of airway-lining fluid and the regulation of airway tone. A non-invasive delivery of therapeutic medicines may be achieved via pulmonary administration, which has a high permeability, a large absorptive surface area (about 70–140 m^2^ in human adults with an exceptionally thin absorptive mucosal membrane), and a good blood supply [277,278,279]. Chlorofluorocarbons propellants are also being phased out of industrial and residential applications across the world, which has sparked the creation of novel substitutes. Various amendments to the Montreal Protocol on Substances that deplete the ozone layer as well as to the original text outline the timescale for this project. As a consequence of these critical modifications, pharmaceutical companies and academics were able to develop new methods of administering drugs through the pulmonary route. Respiratory infection drugs and systemic sickness treatments are separate medication categories [280]. In this second state, the lungs are solely regarded as a route for systemic pharmaceutical administration.

### 11.4. Skin Drug Delivery

Due to physicochemical constraints, the protective function of human skin restricts the types of permeants that may pass through it. Passive skin administration necessitates the use of a lipophilic drugs with a molecular weight below 500 Da. Only a small number of commercially available medications fulfill these strict standards for percutaneous administration. Active and passive strategies have emerged in recent years to improve drug delivery. The passive strategy involves improving the formulation or the medicinal carrier in order to increase skin permeability. Drugs having molecular weights larger than 500 Da, on the other hand, do not benefit from passive techniques. For active techniques, physical or mechanical methods of distribution have been demonstrated to be more effective in the long run [281,282].

## 12. Ongoing Clinical Trials on Natural and Synthetic Biomaterials

This section contains human clinical trials that are currently ongoing with natural and synthetic biomaterials. Here, we highlighted the present stage of a clinical trial of the selected biomaterials. All clinical information we collected from ClinicalTrials.gov websites. Currently, there are several clinical studies ongoing with natural and synthetic biomaterials and their formulations, summarized below in Table 5. Chitosan, a modified carbohydrate polymer obtained from chitin present in various natural sources, has a wide array of applications in the fields of medicine and pharmaceuticals. Its main approved purpose is serving as a biomaterial in medical devices. Additionally, chitosan and its derivatives play essential roles in pharmaceuticals, functioning as excipients, drug carriers, or therapeutic agents. At the moment, chitosan and its composition are in different clinical phases to treat different diseases, such as periodontitis, periodontal pocket, periodontal diseases, periodontal inflammation, osteoarthritis, and vaginal bleeding. Cellulose is a complex polysaccharide that forms the principal component of the plant cell wall and is the most abundant biopolymer in the biological world. Cellulose and microcrystalline cellulose are currently in the early clinical phase to mitigate some diseases, such as long COVID-19, sarcoidosis, and pulmonary diseases. With a collagen sponge and hydrolysed collagen-based supplements, different researchers are trying to find a new way to treat alveolar osteitis, pain (acute and chronic), and knee osteoarthritis. Different formulations of dextran and gelatin are now applied to treat pain (acute and chronic) and knee osteoarthritis, but some of them are in the early clinical phase, and few of the formulations are in phase 4 clinical trials. 

Besides natural biomaterials, synthetic biomaterials are also gaining interest from researchers to treat different types of diseases. Dendrimer, polyethylene glycol, polylactic acid, polycaprolactone, and hydroxy ethyl cellulose are now in different clinical trial phases to treat different diseases, such as amyotrophic lateral sclerosis, type 2 diabetes mellitus, pigmentation disorder, congenital breast defect correction, and osteoarthritis, respectively.

In the below table, we have mentioned some natural and synthetic biomaterials that are currently in the clinical trial; in the table, we highlighted some points: (1) clinical trial phase; (2) participants number; (3) composition or device; (4) diseases or conditions; (5) estimated study completion date.

## 13. Future Direction and Perspectives

More than 1500 patents have been registered in this sector in the last two decades and dozens of clinical investigations have been completed [284]. Natural and synthetic biomaterials seem to be the ideal paradigm for therapy, diagnostics, and generation of drug delivery carriers, as detailed in the sections above. With nanoparticles, it will be easier than other techniques to deliver a precise dose of medicine to cancerous or tumorous cells without interfering with the normal physiology of healthy cells. However, material homogeneity, drug loading, and release capabilities need to be further researched. This review covers vast area to the delivery and diagnosis of medicines using metal-based nanoparticles and synthetic and natural biopolymers. One of the most promising areas of research is the use of precious metals, such as gold and silver, in diagnostic and therapeutic procedures. A major source of excitement in this review is nanoparticles, which seem to be able to penetrate soft tumour tissues, making the tumour accessible to radiation-based heat therapy for targeted eradication.

Natural and synthetic biomaterials are rapidly attracting the attention of researchers who want to use them to administer drugs in a manner that is both precise and regulated. In the near future, these materials are expected to earn greater scientific legitimacy. Despite widespread recognition of their long-term potential, nanomedicine and nano-DDSs have yet to have a significant influence on the healthcare system, notably in cancer treatment and diagnostics. Due to the fact that this field has only been studied for two decades, there are many key fundamental qualities that have yet to be discovered. One of the most important future study areas will be the development of basic indicators of sick tissues, such as vital biological markers, that permit precise targeting without interrupting the normal cellular process. With an increasing understanding of disease mechanisms and the discovery of nanomaterial-subcellular scale indicators, new diagnostic and therapeutic avenues will open up by expanding the use of the nanomedicine field. Because of this, nanomedicine will benefit from an increased understanding of disease molecular signatures.

New studies are needed to maximize the potential of nanomedicine, including animal studies and cross-disciplinary research. The growing need for accurate medications and diagnostics has led to the development of nanorobots and nanodevices for tissue diagnostics and healing systems. However, nanomedicine has drawbacks, such as acute and chronic toxicity effects on humans and the environment, and the regulation of nanomedicine will change with advancements in applications. Nanotechnology has become a significant component in increasing the effectiveness of natural bioactive chemicals, such as berberine, curcumin, resveratrol, and quercetin. Nanocarriers made with gold, silver, and other metals; titanium dioxide polymers; solid lipid nanoparticles; micelles; and superparamagnetic iron oxide nanoparticles have been identified to boost their effectiveness. Natural biomaterials, such as biodegradable, biocompatible, renewable, and low-toxicity biomaterials, are in high demand. Advanced biopolymer research issues include crosslinking biopolymers to make them more resistant to industrial processing and biological matrixes. Polymeric nanoparticles have been produced using solvent evaporation, emulsion polymerization, and surfactant-free emulsion polymerization. Nanomedicine research has seen a spike in interest in integrating therapy and diagnostics, as shown by the cancer disease model. Advances in nanomedicine have allowed for better diagnosis and treatment of illnesses by combining diagnostic and therapeutic processes.

## Figures and Tables

**Table 1:** List of natural and synthetic biomaterials.

Natural Biomaterials	Synthetic Biomaterials
Amylose	Cellulose acetate phthalate
Cellulose	Poly(vinyl acetate)
Chitin	Hydroxy acetate phthalate
Chitosan	Phthalate 55
Pectin	Phthalate 50
Alginate	Eudragit L 100
Dextran	Eudragit RS 30 D
Cyclodextrin	Hydroxy ethyl cellulose
Arginine	Poly(diethyl siloxane) (PDES)
Guar Gum	Poly(methyl hydrogen siloxane) (PMHS)
poly(glycolic acid)	Poly(glycolic acid) (PGA)
poly(lactic acid)	Poly(acrylic acid) (PAAc)
Hyaluronic acid	Poly(lactic acid) (PLA)
Heparin	Poly(lactic acid-*co*-glycolic acid) (PLGA)
Chondroitin sulphate	Poly(2-hydroxyethyl methacrylate)
Agarose	Poly(*N*-isopropyl acrylamide)
Gellan	Polycaprolactone (PCL)
Keratin	Poly(ethylenimine)s
Silk fibroin	Dendritic polymers
Collagen	Poly(*N*,*N*-diethylacrylamide)(PDEAAm)
Gelatin	Poly(ethylene oxide) (PEO)
Fibronectin	Poly(ethylene glycol) (PEG)
Laminins	Poly(2-(methacryloyloxy)ethyl phosphorylcholine)
Elastin	Poly(methyl methacrylate)
Glycosaminoglycan	Poly(maleic anhydride)
Ovomucin	Poly(methacrylate)
Lactoferrin	Poly(vinylacetaldiethylaminoacetate) (PVD)
Sericin	Poly(2-acrylamido 2-methylpropane sulfonate)

**Table 2:** Monomers are extensively utilized to make hydrogels for medical and pharmacological purposes.

Abbreviation	Monomer
HEMA	2-Hydroxyethyl methacrylate
HEEMA	2-Hydroxyethoxyethyl methacrylate
HDEEMA	2-Hydroxydiethoxy methacrylate
MEMA	2-Methoxyethyl methacrylate
EEMA	2-Ethoxyethyl methacrylate
MPC	2-(Methacryloyloxy)ethyl phosphorylcholine
EGDMA	Ethylene glycol dimethacrylate
NVP	*N*-Vinyl-2-pyrrolidine
NIPAAm	*N*-Isopropyl acrylamide
VAc	Vinyl acetate
AAc	Acrylic acid
MAAc	Methacrylic acid
HPMA	*N*-(2-Hydroxypropyl) methacrylamide
EG	Ethylene glycol
PEGA	PEG acrylate
PEGMA	PEG methacrylate
PEGDA	PEG diacrylate
PEGDMA	PEG dimethacrylate

**Table 3:** Common hydrogels used in the pharmaceutical field for the preparation of controlled drug delivery systems.

Hydrogel Polymer	Notes
Biodegradable Hydrogels	
Poly(glycolic acid) (PGA)	
Poly(lactic acid) (PLA)	
PLA-*b*-PGA	
PLA-*b*-PEG	
Chitosan	
Dextran	
Non-biodegradable hydrogels	
Poly(2-hydroxyethyl methacrylate) (PHEMA)	
Poly(vinyl alcohol) (PVA)	
Poly(*N*-vinyl pyrrolidone) (PNVP)	
Poly(ethylene-*co*-vinyl-acetate) (PEVAc)	
Poly(acrylamide) (PAAm)	
Poly(acrylic acid) (PAAc)	pH-responsive
Poly(methacrylic acid) (PMAAc)	pH-responsive
Poly(*N*,*N*-diethylaminoethyl methacylate) (PDEAMEA)	pH-responsive
Poly(*N*,*N*-dimethylaminoethyl methacrylate) (PDMAEMA)	pH-responsive
Poly(methacrylic acid)-*graft*-poly(ethylene glycol) (PMAAc-*g*-PEG))	Complexing hydrogels
Poly(acrylic acid)-*graft*-poly(ethylene glycol) (PAAc-*g*-PEG))	Complexing hydrogels
Poly(*N*-isopropyl acrylamide) (PNIPAAm)	Temperature-responsive
Poly(NIPAAm-*co*-AAc)	pH/temperature-responsive
Poly(NIPAAm-*co*-MAAc)	pH/temperature-responsive

**Table 4:** Different controlled release devices may be used to acquire the diffusional exponent, *n*, as well as the associated drug release processes [246].

Diffusion Exponent, *n*			Drug Release Mechanism
Thin Film	Cylindrical Sample	Spherical Sample	
0.5	0.45	0.43	Fickian diffusion
0.5 < *n* < 1.0	0.45 < *n* < 1.0	0.43 < *n* < 1.0	Anomalous transport (non-Fickian diffusion)
1.0	1.0	1.0	Case II transport (zero-order release)

**Table 5:** Natural and synthetic biomaterials-based formulations or devices currently in clinical trials [283].

No.	Clinical Trial Phase	Participants	Composition or Device	Disease or Conditions	Estimated Study Completion Date
1	Phase 4	130	Chitosan	Vaginal bleeding, loop electrosurgical excision	31 January 2024
2	N/A	40	4% Chitosan gel (pH 3.48).	Periodontitis, periodontal pocket, periodontal diseases, periodontal inflammation	31 December 2024
3	Phase 2	48	α-Mangostin hydrogel film with chitosan alginate base	Recurrent aphthous stomatitis	20 December 2024
4	Phase 4	104	KiOmedine^®^ CM-chitosan (KiOmed Pharma, Herstal, Belgium)	Osteoarthritis, knee	20 February 2024
5	Phase 3	150	Microcrystalline cellulose	long COVID-19	1 November 2025
6	Phase 2	40	Cellulose	Sarcoidosis, pulmonary	1 September 2024
7	Phase 2	40	Collagen sponge	Alveolar osteitis	15 November 2023 (Just completed)
8	N/A	80	Hydrolysed collagen-based supplement	Pain (acute and chronic), knee osteoarthritis	June 2024
9	Phase 4	90	Iron dextran injection	cCKD-chronic kidney disease	31 December 2024
10	N/A	100	Dextran 40	Type 2 diabetes mellitus, diabetic kidney disease	1 September 2026
11	N/A	30	Gelatin sponge-loaded apoptotic vesicle complex (Kuaikang^®^, Hengshui Kuaikang Medical Device Co. Ltd., Hengshui, China)	Third molar extraction	30 December 2024
12	N/A	50	Gelatin sponge stabilization with suture and cyanoacrylate	Wound heal	5 April 2024
13	Phase 1 and 2	26	18F-OP-801 (18F hydroxyl dendrimer)	Amyotrophic lateral sclerosis (ALS)	30 June 2023 (Just completed)
14	Phase 4	480	Poly(ethylene glycol) Losenatide	Type2 diabetes mellitus	31 December 2025
15	Phase 4	102	Poly(ethylene glycol)s	Hepatic encephalopathy	30 December 2023
16	N/A	40	Poly(ethylene glycol) (PEG) mediated fusion	Peripheral nerve injuries	1 October 2024
17	Phase 2	150	Poly(ethylene glycol) (PEG) recombinant human erythropoietin injection	Renal anaemia	
18	N/A	40	3D printed upper-limb prosthesis (made of poly(lactic acid))	Amniotic band syndrome, upper extremity deformities, congenital	31 August 2024
19	N/A	40	PDLLA (Poly(*D*,*L*-lactic acid)	Pigmentation, pigmentation disorder	31 December 2023
20	N/A	50	Gana X (Poly(*L*-lactic acid))	Buttocks volume loss	November 2024
21	N/A	150	Poly(*L*-lactic acid) [PLLA-SCA, Sculptra^®^, Dermik Laboratories, New Jersey, USA]	Skin laxity	November 2023
22	N/A	20	Surgical implantation of the Polycaprolactone (PCL) breast scaffold with autologous fat grafting	Breast implant revision, congenital breast defect correction	20 June 2026
23	N/A	60	PCL based breast implants Lifesil	Breast augmentation, mammoplasty	30 May 2024
24	N/A	50	Hydrogel injection (Hydroxy ethyl cellulose)	Osteoarthritis, knee pain	31 July 2024

## Data Availability

Data will be made available on request.

## Abbreviation

**Abbreviation**	**Definition**
DDS	Drug delivery system
PAE	Poly(amino ester)
IV	Intravenous
5-ALA	5-Aminolevulinic
CC	Colon cancer
CB	Cathepsin B
HA	Hyaluronic acid
Cy5.5	Cyanine 5.5
IRT	Irinotecan
PFH	Perfluorohexane
DOX	Doxorubicin
GdNGs	Gadolinium nanogels
MRI	Magnetic resonance imaging
NIR	Near infrared
C6	Chlorine 6
PTX	Paclitaxel
FA	Folic acid
PHEMA	Poly(2-hydroxyethyl Methacrylate)
HEMA	2-Hydroxyethyl methacrylate
TPGDA	Tripropyleneglycol diacrylate
PNIPAAm	Poly(*N*-isopropyl acrylamide)
LCST	Lower critical solution temperature
AAc	Acrylic acid
PAA	Poly(allyl amine)
PEI	Poly(ethylenimine)
BPEI	Branched poly(ethylenimine)
QDs	Quantum dots
FD	Fucoidan
TMC	Trimethyl chitosan
TCD	Tetracyclododecene
CMC	Critical micelles concentration
FAPPI	Folate acid conjugated poly(propylene imine)
MTX	Methotrexate
XG	Xanthan gum
DCT	Docetaxel
PLGA	poly(*D*,*L*-lactide-*co*-glycolide)
HACE	HA acid–ceramide
PLA	Poly(lactic acid)
PEO	Poly(ethylene oxide)
PPO	Poly(propylene oxide)
RES	Reticuloendothelial system
ABC	Accelerated blood clearance
P-gP	P-glycoprotein
IP	Intraperitoneal
PNVP	Poly(*N*-vinyl pyrrolidone)
BBB	Blood-brain barrier
CNS	Central nervous system
EVs	Extracellular vesicles
MSCs	Mesenchymal stem cells
MVBs	Multivesicular bodies

## References

[1] Patra J.K., Das G., Fraceto L.F., Campos E.V.R., Rodriguez-Torres M.D.P., Acosta-Torres L.S., Diaz-Torres L.A., Grillo R., Swamy M.K., Sharma S. (2018). Nano based drug delivery systems: Recent developments and future prospects. J. Nanobiotechnol..

[2] Daraba O.M., Cadinoiu A.N., Rata D.M., Atanase L.I., Vochita G. (2020). Antitumoral drug-loaded biocompatible polymeric nanoparticles obtained by non-aqueous emulsion polymerization. Polymers.

[3] Jacob J., Haponiuk J.T., Thomas S., Gopi S. (2018). Biopolymer based nanomaterials in drug delivery systems: A review. Mater. Today Chem..

[4] Gopi S., Amalraj A., Sukumaran N.P., Haponiuk J.T., Thomas S. (2018). Biopolymers and Their Composites for Drug Delivery: A Brief Review. Macromol. Symp..

[5] Rodriguez-Galan A., Franco L., Puiggali J. (2011). Degradable Poly(ester amide)s for Biomedical Applications. Polymers.

[6] Giri G., Maddahi Y., Zareinia K. (2021). A Brief Review on Challenges in Design and Development of Nanorobots for Medical Applications. Appl. Sci..

[7] Al-Arif S., Quader N., Shaon A.M., Islam K.K. (2011). Sensor based autonomous medical nanorobots: A cure to demyelination. J. Sel. Areas Nanotechnol..

[8] Mamani J.B., Borges J.P., Rossi A.M., Gamarra L.F. (2023). Magnetic Nanoparticles for Therapy and Diagnosis in Nanomedicine. Pharmaceutics.

[9] Kim J., Cho H., Lim D.K., Joo M.K., Kim K. (2023). Perspectives for Improving the Tumor Targeting of Nanomedicine via the EPR Effect in Clinical Tumors. Int. J. Mol. Sci..

[10] Dhaliwal A., Zheng G. (2019). Improving accessibility of EPR-insensitive tumor phenotypes using EPR-adaptive strategies: Designing a new perspective in nanomedicine delivery. Theranostics.

[11] Herea D.D., Zară-Dănceanu C.M., Lăbușcă L., Minuti A.E., Stavilă C., Ababei G., Tibu M., Grigoraș M., Lostun M., Stoian G. (2023). Enhanced Multimodal Effect of Chemotherapy, Hyperthermia and Magneto-Mechanic Actuation of Silver-Coated Magnetite on Cancer Cells. Coatings.

[12] Lu H., Wang J., Wang T., Zhong J., Bao Y., Hao H. (2016). Recent progress on nano- structures for drug delivery applications. J. Nanomater..

[13] Blanco E., Shen H., Ferrari M. (2015). Principles of nanoparticle design for overcoming biological barriers to drug delivery. Nat. Biotechnol..

[14] Kumari A., Kumar V., Yadav S. (2012). Nanotechnology: A tool to enhance therapeutic values of natural plant products. Trends Med. Res..

[15] Guanyou L., Miqin Z. (2023). Ligand Chemistry in Antitumor Theranostic Nanoparticles. Acc. Chem. Res..

[16] Haozhe H., Xindan Z., Lihua D., Minwen Y., Yonglai L., Jiajia X., Jun W., Xintao S. (2022). Molecular imaging nanoprobes for theranostic applications. Adv. Drug Deliv. Rev..

[17] Baroni S., Argenziano M., La Cava F., Soster M., Garello F., Lembo D., Cavalli R., Terreno E. (2023). Hard-Shelled Glycol Chitosan Nanoparticles for Dual MRI/US Detection of Drug Delivery/Release: A Proof-of-Concept Study. Nanomaterials.

[18] Lee C.M., Jang D., Kim J., Cheong S.J., Kim E.M., Jeong M.H., Kim S.H., Kim D.W., Lim S.T., Sohn M.H. (2011). Oleyl-Chitosan nanoparticles based on a dual probe for Optical/MR imaging in vivo. Bioconjug. Chem..

[19] Yang S.J., Lin F.H., Tsai H.M., Lin C.F., Chin H.C., Wong J.M., Shieh M.J. (2011). Alginate-folic acid-modified chitosan nanoparticles for photodynamic detection of intestinal neoplasms. Biomaterials.

[20] Ryu J.H., Na J.H., Ko H.K., You D.G., Park S., Jun E., Yeom H.J., Seo D.H., Park J.H., Jeong S.Y. (2014). Non-invasive optical imaging of cathepsin B with activatable fluorogenic nanoprobes in various metastatic models. Biomaterials.

[21] Juhaščik M., Kováčik A., Huerta-Ángeles G. (2022). Recent Advances of Hyaluronan for Skin Delivery: From Structure to Fabrication Strategies and Applications. Polymers.

[22] Puluhulawa L.E., Joni I.M., Elamin K.M., Mohammed A.F.A., Muchtaridi M., Wathoni N. (2022). Chitosan–Hyaluronic Acid Nanoparticles for Active Targeting in Cancer Therapy. Polymers.

[23] Uthappa U.T., Suneetha M., Ajeya K.V., Ji S.M. (2023). Hyaluronic Acid Modified Metal Nanoparticles and Their Derived Substituents for Cancer Therapy: A Review. Pharmaceutics.

[24] Tang H., Zhang Z., Zhu M., Xie Y., Lv Z., Liu R., Shen Y., Pei J. (2023). Efficient Delivery of Gemcitabine by Estrogen Receptor-Targeted PEGylated Liposome and Its Anti-Lung Cancer Activity In Vivo and In Vitro. Pharmaceutics.

[25] Wang G., Gao S., Tian R., Miller-Kleinhenz J., Qin Z., Liu T., Li L., Zhang F., Ma Q., Zhu L. (2018). Theranostic hyaluronic acid-iron micellar nanoparticles for magnetic-field-enhanced in vivo cancer chemotherapy. Chem. Med. Chem..

[26] Ma S., Kim J.H., Chen W., Li L., Lee J., Xue J., Liu Y., Chen G., Tang B., Tao W. (2023). Cancer Cell-Specific Fluorescent Prodrug Delivery Platforms. Adv. Sci..

[27] Weng Y., Yang G., Li Y., Xu L., Chen X., Song H., Zhao C.-X. (2023). Alginate-based materials for enzyme encapsulation. Adv. Colloid. Interface Sci..

[28] Baghbani F., Moztarzadeh F., Mohandesi J.A., Yazdian F., Mokhtari-Dizaji M. (2016). Novel alginate-stabilized doxorubicin-loaded nanodroplets for ultrasounic theranosis of breast cancer. Int. J. Biol. Macromol..

[29] Podgórna K., Szczepanowicz K., Piotrowski M., Gajdošová M., Štěpánek F., Warszyński P. (2017). Gadolinium alginate nanogels for theranostic applications. Coll. Surf. B.

[30] Ding Z., Liu P., Hu D., Sheng Z., Yi H., Gao G., Wu Y., Zhang P., Ling S., Cai L. (2017). Redox-responsive dextran based theranostic nanoparticles for near-infrared/magnetic resonance imaging and magnetically targeted photodynamic therapy. Biomater. Sci..

[31] Ali M.M., Brown S.L., Snyder J.M. (2021). Dendrimer-Based Nanomedicine (Paramagnetic Nanoparticle, Nanocombretastatin, Nanocurcumin) for Glioblastoma Multiforme Imaging and Therapy. Nov. Aproaches Cancer Study.

[32] Kobryń J., Raszewski B., Zięba T., Musiał W. (2023). Modified Potato Starch as a Potential Retardant for Prolonged Release of Lidocaine Hydrochloride from Methylcellulose Hydrophilic Gel. Pharmaceutics.

[33] Nayak P., Bentivoglio V., Varani M., Signore A. (2023). Three-Dimensional In Vitro Tumor Spheroid Models for Evaluation of Anticancer Therapy: Recent Updates. Cancers.

[34] Simeonov M., Kostova B., Vassileva E. (2023). Interpenetrating Polymer Networks of Poly(2-hydroxyethyl methacrylate) and Poly(*N***,***N*-dimethylacrylamide) as Potential Systems for Dermal Delivery of Dexamethasone Phosphate. Pharmaceutics.

[35] Hou X., Li J., Hong Y., Ruan H., Long M., Feng N., Zhang Y. (2023). Advances and Prospects for Hydrogel-Forming Microneedles in Transdermal Drug Delivery. Biomedicines.

[36] Hanak B.W., Hsieh C.Y., Donaldson W., Browd S.R., Lau K.S., Shain W. (2018). Reduced cell attachment to poly(2-hydroxyethyl methacrylate)-coated ventricular catheters in vitro. J. Biomed. Mater. Res. B Appl. Biomater..

[37] Liu Z., Zhang S., Gao C., Meng X., Wang S., Kong F. (2022). Temperature/pH-Responsive Carboxymethyl Cellulose/Poly(*N*-isopropyl acrylamide) Interpenetrating Polymer Network Aerogels for Drug Delivery Systems. Polymers.

[38] Szewczyk-Łagodzińska M., Plichta A., Dębowski M., Kowalczyk S., Iuliano A., Florjańczyk Z. (2023). Recent Advances in the Application of ATRP in the Synthesis of Drug Delivery Systems. Polymers.

[39] Luo S., Lv Z., Yang Q., Chang R., Wu J. (2023). Research Progress on Stimulus-Responsive Polymer Nanocarriers for Cancer Treatment. Pharmaceutics.

[40] Jente V., Tomáš S., Valentin V.J., Yann B., Joachim F.R., Van G., Richard H. (2023). Poly(*N*-allyl acrylamide) as a Reactive Platform toward Functional Hydrogels. ACS Macro. Lett..

[41] Sarabia-Vallejos M.A., Cerda-Iglesias F.E., Pérez-Monje D.A., Acuña-Ruiz N.F., Terraza-Inostroza C.A., Rodríguez-Hernández J., González-Henríquez C.M. (2023). Smart Polymer Surfaces with Complex Wrinkled Patterns: Reversible, Non-Planar, Gradient, and Hierarchical Structures. Polymers.

[42] Yoo M.K., Sung Y.K., Lee Y.M., Cho C.S. (2000). Effect of polyelectrolyte on the lower critical solution temperature of poly(*N*-isopropyl acrylamide) in the poly(NIPAAm-*co*-acrylic acid) hydrogel. Polymer.

[43] Zhu Y., Liu C., Pang Z. (2019). Dendrimer-Based Drug Delivery Systems for Brain Targeting. Biomolecules.

[44] Corchero J.L., Favaro M.T.P., Márquez-Martínez M., Lascorz J., Martínez-Torró C., Sánchez J.M., López-Laguna H., de Souza Ferreira L.C., Vázquez E., Ferrer-Miralles N. (2023). Recombinant Proteins for Assembling as Nano-and Micro-Scale Materials for Drug Delivery: A Host Comparative Overview. Pharmaceutics.

[45] Madaan K., Kumar S., Poonia N., Lather V., Pandita D. (2014). Dendrimers in drug delivery and targeting: Drug-dendrimer interactions and toxicity issues. J. Pharm. Bioallied Sci..

[46] Noriega-Luna B., Godínez L.A., Rodríguez F.J., Rodríguez A., Larrea G.Z.L.D., Sosa-Ferreyra C.F., Mercado-Curiel R.F., Manríquez J., Bustos E.B. (2014). Applications of Dendrimers in Drug Delivery Agents, Diagnosis, Therapy, and Detection. J. Nanomater..

[47] Sahiner M., Yilmaz A.S., Demirci S., Sahiner N. (2023). Physically and Chemically Crosslinked, Tannic Acid Embedded Linear PEI-Based Hydrogels and Cryogels with Natural Antibacterial and Antioxidant Properties. Biomedicines.

[48] Cai X., Dou R., Guo C., Tang J., Li X., Chen J., Zhang J. (2023). Cationic Polymers as Transfection Reagents for Nucleic Acid Delivery. Pharmaceutics.

[49] Tarach P., Janaszewska A. (2021). Recent Advances in Preclinical Research Using PAMAM Dendrimers for Cancer Gene Therapy. Int. J. Mol. Sci..

[50] Ortega M.Á., Guzmán Merino A., Fraile-Martínez O., Recio-Ruiz J., Pekarek L., Guijarro L.G., García-Honduvilla N., Álvarez-Mon M., Buján J., García-Gallego S. (2020). Dendrimers and Dendritic Materials: From Laboratory to Medical Practice in Infectious Diseases. Pharmaceutics.

[51] Sung Y.K., Kim S.W. (2020). Recent advances in polymeric drug delivery systems. Biomater. Res..

[52] Assad H., Assad A., Kumar A. (2023). Recent Developments in 3D Bio-Printing and Its Biomedical Applications. Pharmaceutics.

[53] Lee T.S., Bee S.T. (2019). A practical guide for the processing, manufacturing and applications of PLA. Polylactic Acid.

[54] Heller J., Barr J., Yng S., Abdellauoi K.S., Gurny R. (2002). Poly(ortho esters): Synthesis, characterization, properties and uses. Adv. Drug Deliv. Rev..

[55] Kumar N., Langer R.S., Domb A.J. (2002). Polyanhydrides: An overview. Adv. Drug Deliv. Rev..

[56] Kamaly N., Yameen B., Wu J., Farokhzad O.C. (2016). Degradable controlled-release polymers and polymeric nanoparticles: Mechanisms of controlling drug release. Chem. Rev..

[57] Mustafai A., Zubair M., Hussain A., Ullah A. (2023). Recent Progress in Proteins-Based Micelles as Drug Delivery Carriers. Polymers.

[58] Xu W., Ling P., Zhang T. (2013). Polymeric micelles, a promising drug delivery system to enhance the bioavailability of poorly water-soluble drugs. J. Drug Deliv..

[59] Kotta S., Aldawsari H.M., Badr-Eldin S.M., Nair A.B., YT K. (2022). Progress in Polymeric Micelles for Drug Delivery Applications. Pharmaceutics.

[60] Devarajan P.V., Jain S. (2015). Targeted Drug Delivery: Concepts and Design.

[61] Hu Q., Lu Y., Luo Y. (2021). Recent advances in dextran-based drug delivery systems: From fabrication strategies to applications. Carbohydr. Polym..

[62] Wakaskar R.R. (2017). Polymeric micelles for drug delivery. Int. J. Drug Dev. Res..

[63] Mandal A., Bisht R., Rupenthal I.D., Mitra A.K. (2017). Polymeric micelles for ocular drug delivery: From structural frameworks to recent preclinical studies. J. Control. Release.

[64] Kesharwani P., Xie L., Banerjee S., Mao G., Padhye S., Sarkar F.H., Iyer A.K. (2015). Hyaluronic acid-conjugated polyamidoamine dendrimers for targeted delivery of 3,4-difluorobenzylidene curcumin to CD44 overexpressing pancreatic cancer cells. Coll. Surf. B.

[65] Kesharwani P., Jain K., Jain N.K. (2014). Dendrimer as nanocarrier for drug delivery. Progr. Polym. Sci..

[66] Jain K., Gupta U., Jain N.K. (2014). Dendronized nanoconjugates of lysine and folate for treatment of cancer. Eur. J. Pharm. Biopharm..

[67] Kaur A., Jain K., Mehra N.K., Jain N. (2017). Development and characterization of surface engineered PPI dendrimers for targeted drug delivery. Artif. Cells Nanomed. Biotechnol..

[68] Choi S.J., Lee J.K., Jeong J., Choy J.H. (2013). Toxicity evaluation of inorganic nanoparticles: Considerations and challenges. Mol. Cell Toxicol..

[69] Kong F.Y., Zhang J.W., Li R.F., Wang Z.X., Wang W.J., Wang W. (2017). Unique roles of gold nanoparticles in drug delivery, targeting and imaging applications. Molecules.

[70] Volkov Y. (2015). Quantum dots in nanomedicine: Recent trends, advances and unresolved issues. Biochem. Biophys. Res. Commun..

[71] Linkova N., Diatlova A., Zinchenko Y., Kornilova A., Snetkov P., Morozkina S., Medvedev D., Krasichkov A., Polyakova V., Yablonskiy P. (2023). Pulmonary Sarcoidosis: Experimental Models and Perspectives of Molecular Diagnostics Using Quantum Dots. Int. J. Mol. Sci..

[72] Prusty K., Swain S.K. (2018). Nano silver decorated polyacrylamide/dextran nanohydrogels hybrid composites for drug delivery applications. Mater. Sci. Eng..

[73] Marcu A., Pop S., Dumitrache F., Mocanu M., Niculite C., Gherghiceanu M., Lungu C., Fleaca C., Ianchis R., Barbut A. (2013). Magnetic iron oxide nanoparticles as drug delivery system in breast cancer. Appl. Surf. Sci..

[74] Junyaprasert V.B., Morakul B. (2015). Nanocrystals for enhancement of oral bioavailability of poorly water-soluble drugs. Asian J. Pharm. Sci..

[75] Du J., Li X., Zhao H., Zhou Y., Wang L., Tian S., Wang Y. (2015). Nanosuspensions of poorly water-soluble drugs prepared by bottom-up technologies. Int. J. Pharm..

[76] Ni R., Zhao J., Liu Q., Liang Z., Muenster U., Mao S. (2017). Nanocrystals embedded in chitosan-based respirable swellable microparticles as dry powder for sustained pulmonary drug delivery. Eur. J. Pharm. Sci..

[77] McNamara K., Tofail S.A. (2017). Nanoparticles in biomedical applications. Adv. Phys..

[78] Xu G., Zeng S., Zhang B., Swihart M.T., Yong K.T., Prasad P.N. (2016). New generation cadmium-free quantum dots for biophotonics and nanomedicine. Chem. Rev..

[79] Shi Y., Pramanik A., Tchounwou C., Pedraza F., Crouch R.A., Chavva S.R., Vangara A., Sinha S.S., Jones S., Sardar D. (2015). Multifunctional biocompatible graphene oxide quantum dots decorated magnetic nanoplatform for efficient capture and two-photon imaging of rare tumor cells. ACS Appl. Mater. Interfaces.

[80] Ahmad J., Garg A., Mustafa G., Ahmad M.Z., Aslam M., Mishra A. (2023). Hybrid Quantum Dot as Promising Tools for Theranostic Application in Cancer. Electronics.

[81] Zheng F.F., Zhang P.H., Xi Y., Chen J.J., Li L.L., Zhu J.J. (2015). Aptamer/graphene quantum dots nanocomposite capped fluorescent mesoporous silica nanoparticles for intracellular drug delivery and real-time monitoring of drug release. Anal. Chem..

[82] Huang C.L., Huang C.C., Mai F.D., Yen C.L., Tzing S.H., Hsieh H.T., Ling Y.C., Chang J.Y. (2015). Application of paramagnetic graphene quantum dots as a platform for simultaneous dual-modality bioimaging and tumor targeted drug delivery. J. Mater. Chem. B.

[83] Olerile L.D., Liu Y., Zhang B., Wang T., Mu S., Zhang J., Selotlegeng L., Zhang N. (2017). Near-infrared mediated quantum dots and paclitaxel co-loaded nanostructured lipid carriers for cancer theragnostic. Coll. Surf. B.

[84] Cai X., Luo Y., Zhang W., Du D., Lin Y. (2016). pH-Sensitive ZnO quantum dots– doxorubicin nanoparticles for lung cancer targeted drug delivery. ACS Appl. Mater. Interfaces.

[85] Balaji A.B., Pakalapati H., Khalid M., Walvekar R., Siddiqui H., Shimpi N.G. (2017). Natural and synthetic biocompatible and biodegradable polymers. Biodegradable and Biocompatible Polymer Composites: Processing, Properties and Applications.

[86] Bassas-Galia M., Follonier S., Pusnik M., Zinn M. (2017). Natural polymers: A source of inspiration. Bioresorbable Polymers for Biomedical Applications.

[87] Lohcharoenkal W., Wang L., Chen Y.C., Rojanasakul Y. (2014). Protein nanoparticles as drug delivery carriers for cancer therapy. BioMed. Res. Int..

[88] Cardoso M.J., Costa R.R., Mano J.F. (2016). Marine origin polysaccharides in drug delivery systems. Mar. Drugs.

[89] Yu Z., Yu M., Zhang Z., Hong G., Xiong Q. (2014). Bovine serum albumin nanoparticles as controlled release carrier for local drug delivery to the inner ear. Nanoscale Res. Lett..

[90] Wang B., Wang S., Zhang Q., Deng Y., Li X., Peng L., Zuo X., Piao M., Kuang X., Sheng S. (2019). Recent advances in polymer-based drug delivery systems for local anesthetics. Acta Biomater..

[91] Ewart D., Peterson E.J., Steer C.J. (2019). A new era of genetic engineering for autoimmune and inflammatory diseases. Semin. Arthritis Rheum..

[92] Shamsi M., Mohammadi A., Manshadi M.K.D., Sanati-Nezhad A. (2019). Mathematical and computational modeling of nano-engineered drug delivery systems. J. Control. Release.

[93] Pal K., Sarkar P., Anis A., Wiszumirska K., Jarzębski M. (2021). Polysaccharide-Based Nanocomposites for Food Packaging Applications. Materials.

[94] Su C., Liu Y., Li R., Wu W., Fawcett J.P., Gu J. (2019). Absorption, distribution, metabolism and excretion of the biomaterials used in nanocarrier drug delivery systems. Adv. Drug Deliv. Rev..

[95] Jiang W.Z., Cai Y., Li H.Y. (2017). Chitosan-based spray-dried mucoadhesive microspheres for sustained oromucosal drug delivery. Powder Technol..

[96] Rassu G., Gavini E., Jonassen H., Zambito Y., Fogli S., Breschi M.C., Giunchedi P. (2009). New chitosan derivatives for the preparation of rokitamycin loaded microspheres designed for ocular or nasal administration. J. Pharm. Sci..

[97] Wang F., Zhang Q., Li X., Huang K., Shao W., Yao D., Huang C. (2019). Redox-responsive blend hydrogel films based on carboxymethyl cellulose/chitosan microspheres as dual delivery carrier. Int. J. Biol. Macromol..

[98] Chu L., Zhang Y., Feng Z., Yang J., Tian Q., Yao X., Zhao X., Tan H., Chen Y. (2019). Synthesis and application of a series of amphipathic chitosan derivatives and the corresponding magnetic nanoparticle-embedded polymeric micelles. Carbohydr. Polym..

[99] Qu G., Hou S., Qu D., Tian C., Zhu J., Xue L., Ju C., Zhang C. (2019). Self-assembled micelles based on *N*-octyl-*N*′-phthalyl-*O*-phosphoryl chitosan derivative as an effective oral carrier of paclitaxel. Carbohydr. Polym..

[100] Cuggino J.C., Blanco E.R.O., Gugliotta L.M., Alvarez Igarzabal C.I., Calderon M. (2019). Crossing biological barriers with nanogels to improve drug delivery performance. J. Control. Release.

[101] Li S., Hu L., Li D., Wang X., Zhang P., Wang J., Yan G., Tang R. (2019). Carboxymethyl chitosan-based nanogels via acid-labile ortho ester linkages mediated enhanced drug delivery. Int. J. Biol. Macrmol..

[102] Wang J., Xu M., Cheng X., Kong M., Liu Y., Feng C., Chen X. (2016). Positive/negative surface charge of chitosan based nanogels and its potential influence on oral insulin delivery. Carbohydr. Polym..

[103] Bulbul Y.E., Eskitoros-Togay S.M., Demirtas-Korkmaz F., Dilsiz N. (2019). Multi-walled carbon nanotube-incorporating electrospun composite fibrous mats for controlled drug release profile. Int. J. Pharm..

[104] Ozlu B., Kabay G., Bocek I., Yilmaz M., Piskin A.K., Shim B.S., Mutlu M. (2019). Controlled release of doxorubicin from polyethylene glycol functionalized melanin nanoparticles for breast cancer therapy: Part I. Production and drug release performance of the melanin nanoparticles. Int. J. Pharm..

[105] Gajendiran M., Jo H., Kim K., Balasubramanian S. (2019). In vitro controlled release of tuberculosis drugs by amphiphilic branched copolymer nanoparticles. J. Ind. Eng. Chem..

[106] Safdar R., Omar A.A., Arunagiri A., Regupathi I., Thanabalan M. (2019). Potential of Chitosan and its derivatives for controlled drug release applications—A review. J. Drug Deliv. Sci. Technol..

[107] Bajracharya R., Song J.G., Back S.Y., Han H.-K. (2019). Recent Advancements in Non-Invasive Formulations for Protein Drug Delivery. Comput. Struct. Biotechnol. J..

[108] Lee S.H., Song J.G., Han H.K. (2019). Development of pH-responsive organic-inorganic hybrid nanocomposites as an effective oral delivery system of protein drugs. J. Control. Release.

[109] Du Z., Liu J., Zhang T., Yu Y., Zhang Y., Zhai J., Huang H., Wei S., Ding L., Liu B. (2019). A study on the preparation of chitosan-tripolyphosphate nanoparticles and its entrapment mechanism for egg white derived peptides. Food Chem..

[110] Rekha M.R., Sharma C.P. (2009). Synthesis and evaluation of lauryl succinyl chitosan particles towards oral insulin delivery and absorption. J. Control. Release.

[111] Tsai L.C., Chen C.H., Lin C.W., Ho Y.C., Mi F.L. (2019). Development of multifunctional nanoparticles self-assembled from trimethyl chitosan and fucoidan for enhanced oral delivery of insulin. Int. J. Biol. Macromol..

[112] Trivedi A., Hoffman J., Arora R. (2019). Gene therapy for atrial fibrillation—How close to clinical implementation?. Int. J. Cardiol..

[113] Gollomp K.L., Doshi B.S., Arruda V.R. (2019). Gene therapy for hemophilia: Progress to date and challenges moving forward. Transfus. Apher. Sci..

[114] Gallego I., Villate-Beitia I., Martinez-Navarrete G., Menendez M., Lopez-Mendez T., Soto-Sanchez C., Zarate J., Puras G., Fernandez E., Pedraz J.L. (2019). Non-viral vectors based on cationic niosomes and minicircle DNA technology enhance gene delivery efficiency for biomedical applications in retinal disorders. Nanomedicine.

[115] Kochhar S., Excler J.L., Bok K., Gurwith M., McNeil M.M., Seligman S.J., Khuri-Bulos N., Klug B., Laderoute M., Robertson J.S. (2019). Brighton Collaboration Viral Vector Vaccines Safety Working, G. Defining the interval for monitoring potential adverse events following immunization (AEFIs) after receipt of live viral vectored vaccines. Vaccine.

[116] Mashal M., Attia N., Martinez-Navarrete G., Soto-Sanchez C., Fernandez E., Grijalvo S., Eritja R., Puras G., Pedraz J.L. (2019). Gene delivery to the rat retina by non-viral vectors based on chloroquine-containing cationic niosomes. J. Control. Release.

[117] Massaro M., Barone G., Biddeci G., Cavallaro G., Di Blasi F., Lazzara G., Nicotra G., Spinella C., Spinelli G., Riela S. (2019). Halloysite nanotubes-carbon dots hybrids multifunctional nanocarrier with positive cell target ability as a potential non-viral vector for oral gene therapy. J. Colloid. Interface Sci..

[118] Javan B., Atyabi F., Shahbazi M. (2018). Hypoxia-inducible bidirectional shRNA expression vector delivery using PEI/chitosan-TBA copolymers for colorectal Cancer gene therapy. Life Sci..

[119] Jaiswal S., Dutta P.K., Kumar S., Koh J., Pandey S. (2019). Methyl methacrylate modified chitosan: Synthesis, characterization and application in drug and gene delivery. Carbohydr. Polym..

[120] Mallick S., Song S.J., Bae Y., Choi J.S. (2019). Self-assembled nanoparticles composed of glycol chitosan-dequalinium for mitochondria-targeted drug delivery. Int. J. Biol. Macromol..

[121] Tang Y., Liu Y., Xie Y., Chen J., Dou Y. (2020). Apoptosis of A549 cells by small interfering RNA targeting survivin delivery using poly-β-amino ester/guanidinylated O-carboxymethyl chitosan nanoparticles. Asian J. Pharm. Sci..

[122] Wen L., Hu Y., Meng T., Tan Y., Zhao M., Dai S., Yuan H., Hu F. (2019). Redox-responsive polymer inhibits macrophages uptake for effective intracellular gene delivery and enhanced cancer therapy. Colloids Surf. B.

[123] Lin J.T., Liu Z.K., Zhu Q.L., Rong X.H., Liang C.L., Wang J., Ma D., Sun J., Wang G.H. (2017). Redox-responsive nanocarriers for drug and gene co-delivery based on chitosan derivatives modified mesoporous silica nanoparticles. Colloids Surf. B.

[124] Augst A.D., Kong H.J., Mooney D.J. (2006). Alginate hydrogels as biomaterials. Macromol. Biosci..

[125] Smidsrød O., Skja G. (1990). Alginate as immobilization matrix for cells. Trends Biotechnol..

[126] Chen C.Y., Ke C.J., Yen K.C., Hsieh H.C., Sun J.S., Lin F.H. (2015). 3D Porous Calcium-Alginate Scaffolds Cell Culture System Improved Human Osteoblast Cell Clusters for Cell Therapy. Theranostics.

[127] Doniparthi J., Chappidi S.R., Bhargav E. (2023). Alginate Based Micro Particulate Systems for Drug Delivery, Alginate Biomaterial: Drug Delivery Strategies and Biomedical Engineering.

[128] Alvarez-Lorenzo C., Blanco-Fernandez B., Puga A.M., Concheiro A. (2013). Crosslinked ionic polysaccharides for stimuli-sensitive drug delivery. Adv. Drug Deliv. Rev..

[129] Li Y., Xu Z., Wang J., Pei X., Chen J., Wan Q. (2023). Alginate-based biomaterial-mediated regulation of macrophages in bone tissue engineering. Int. J. Biol. Macromol..

[130] Darrabie M.D., Kendall W.F., Opara E.C. (2006). Effect of alginate composition and gelling cation on micro-bead swelling. J. Microencapsul..

[131] Patil J.S. (2015). Hydrogel system: An approach for drug delivery modulation. Adv. Pharmacoepidemiol. Drug Saf..

[132] Dalheim M.Ø., Vanacker J., Najmi M.A., Aachmann F.L., Strand B.L., Christensen B.E. (2016). Efficient functionalization of alginate biomaterials. Biomaterials.

[133] Yang J.S., Xie Y.J., He W. (2011). Research progress on chemical modification of alginate: A review. Carbohydr. Polym..

[134] Bu H., Kjøniksen A.L., Elgsaeter A., Nyström B. (2006). Interaction of unmodified and hydrophobically modified alginate with sodium dodecyl sulfate in dilute aqueous solution: Calorimetric, rheological, and turbidity studies. Colloids Surf. A Physicochem. Eng..

[135] Gomez C.G., Chambat G., Heyraud A., Villar M., Auzély-Velty R. (2006). Synthesis and characterization of a *β*-CD-alginate conjugate. Polymer.

[136] Pandey S., Mishra S.B. (2011). Graft copolymerization of ethyl acrylate onto xanthan gum, using potassium peroxydisulfate as an initiator. Int. J. Biol. Macromol..

[137] Rana V., Rai P., Tiwary A.K., Singh R.S., Kennedy J.F., Knill C.J. (2011). Modified gums: Approaches and applications in drug delivery. Carbohydr. Polym..

[138] Adepu S., Ramakrishna S. (2021). Controlled Drug Delivery Systems: Current Status and Future Directions. Molecules.

[139] Dai L., Si C. (2019). Recent advances on cellulose-based nano-drug delivery systems: Design of prodrugs and nanoparticles. Curr. Med. Chem..

[140] Sun B., Zhang M., Shen J., He Z., Fatehi P., Ni Y. (2019). Applications of cellulose-based materials in sustained drug delivery systems. Curr. Med. Chem..

[141] Varan G., Benito J.M., Mellet C.O., Bilensoy E. (2017). Development of polycationic amphiphilic cyclodextrin nanoparticles for anticancer drug delivery. Beilstein J. Nanotechnol..

[142] Elmowafy E.M., Tiboni M., Soliman M.E. (2019). Biocompatibility, biodegradation and biomedical applications of poly(lactic acid)/poly(lactic-*co*-glycolic acid) micro and nanoparticles. J. Pharm. Investig..

[143] Yiye L., Coates G.W. (2023). Pairing-Enhanced Regioselectivity: Synthesis of Alternating Poly(lactic-*co*-glycolic acid) from Racemic Methyl-Glycolide. J. Am. Chem. Soc..

[144] Andrade A.L., Fabris J.D., Pereira M.C., Domingues R.Z., Ardisson J.D. (2012). Preparation of composite with silica-coated nanoparticles of iron oxide spinels for applications based on magnetically induced hyperthermia. Hyperfine Interact..

[145] Vieira S., Vial S., Reis R.L., Oliveira J. (2017). Nanoparticles for bone tissue engineering. Biotechnol. Prog..

[146] Hickey J.W., Santos J.L., Williford J.M., Mao H.Q. (2015). Control of polymeric nanoparticle size to improve therapeutic delivery. J. Control. Release.

[147] Banik B.L., Fattahi P., Brown J.L. (2016). Polymeric nanoparticles: The future of nanomedicine. Wiley Interdiscip. Rev. Nanomed. Nanobiotechnol..

[148] Capasso P.U., Maraldi M., Manfredini N., Moscatelli D. (2018). Zwitterionic polyester-based nanoparticles with tunable size, polymer molecular weight, and degradation time. Biomacromolecules.

[149] Sousa F., Fonte P., Cruz A., Kennedy P.J., Pinto I.M., Sarmento B. (2018). Polyester-based nanoparticles for the encapsulation of monoclonal antibodies. Methods Mol. Biol..

[150] Fonte P., Sousa F. (2018). Sarmento B/ Polyester-Based Nanoparticles for Delivery of Therapeutic Proteins. Methods Mol. Biol..

[151] Rijt S.V., Habibovic P. (2017). Enhancing regenerative approaches with nanoparticles. J. R. Soc. Interface.

[152] Motta A.C., Duek E.A.D.R. (2014). Synthesis and characterization of a novel terpolymer based on *L*-lactide, *D*,*L*-lactide and trimethylene carbonate. Mat. Res..

[153] Messias A.D., Martins K.F., Motta A.C., Duek E.A.D.R. (2014). Synthesis, characterization, and osteoblastic cell culture of poly(*L*-*co*-*D*,*L*-lactide-*co*-trimethylene carbonate) scaffolds. Int. J. Biomater..

[154] Cardoso T.P., Ursolino A.P.S., Casagrande P.D.M., Caetano E.B., Mistura D.V., Duek E.A.D.R. (2016). In vivo evaluation of porous hydrogel pins to fill osteochondral defects in rabbits. Rev. Bras. Ortop..

[155] Wang N., Guan Y., Yang L., Jia L., Wei X., Liu H., Guo C. (2013). Magnetic nanoparticles (MNPs) covalently coated by PEO-PPO-PEO block copolymer for drug delivery. J. Colloid. Interface Sci..

[156] Miladi K., Sfar S., Fessi H., Elaissari A. (2016). Nanoprecipitation Process: From Particle Preparation to In Vivo Applications. Polymer Nanoparticles for Nanomedicines.

[157] Wissink J., Herlina H. (2023). Surface-temperature-induced Marangoni effects on developing buoyancy-driven flow. J. Fluid. Mech..

[158] Mahsa M., Mozhdeh S., Hossein H.S. (2023). Thermally driven Marangoni effects on the spreading dynamics of droplets. Int. J. Multiph. Flow..

[159] Beck-Broichsitter M., Erik R., Tobias L., Xiaoying W., Thomas K. (2010). Preparation of nanoparticles by solvent displacement for drug delivery: A shift in the “ouzo region” upon drug loading. Eur. J. Pharm. Sci..

[160] Schubert S., Delaney J.J.T., Schubert U.S. (2011). Nanoprecipitation and nanoformulation of polymers: From history to powerful possibilities beyond poly(lactic acid). Soft Matter..

[161] Prakobvaitayakit M., Nimmannit U. (2003). Optimization of polylactic-*co*-glycolic acid nanoparticles containing itraconazole using 23 factorial design. Pharm. Sci. Tech..

[162] Jan A.T., Azam M., Siddiqui K., Ali A., Choi I., Haq Q.M.R. (2015). Heavy Metals and Human Health: Mechanistic Insight into Toxicity and Counter Defense System of Antioxidants. Int. J. Mol. Sci..

[163] Jabeen N., Muhammad A. (2023). Polysaccharides based biopolymers for biomedical applications: A review. Polym. Adv. Technol..

[164] Mondal A., Nayak A.K., Chakraborty P., Banerjee S., Nandy B.C. (2023). Natural Polymeric Nanobiocomposites for Anti-Cancer Drug Delivery Therapeutics: A Recent Update. Pharmaceutics.

[165] Haisheng H., Yi L., Jianping Q., Quangang Z., Zhongjian C., Wei W. (2019). Adapting liposomes for oral drug delivery. Acta. Pharm. Sin. B.

[166] Allen T.M. (2002). Ligand-Targeted Therapeutics in Anticancer Therapy. Nat. Rev. Cancer.

[167] Riaz M.K., Riaz M.A., Zhang X., Lin C., Wong K.H., Chen X., Zhang G., Lu A., Yang Z. (2018). Surface Functionalization and Targeting Strategies of Liposomes in Solid Tumor Therapy: A Review. Int. J. Mol. Sci..

[168] Millard M., Yakavets I., Zorin V., Kulmukhamedova A., Marchal S., Bezdetnaya L. (2017). Drug delivery to solid tumors: The predictive value of the multicellular tumor spheroid model for nanomedicine screening. Int. J. Nanomed..

[169] Yoo J., Park C., Yi G., Lee D., Koo H. (2019). Active Targeting Strategies Using Biological Ligands for Nanoparticle Drug Delivery Systems. Cancers.

[170] Yu X., Yu-Ping Y., Dikici E., Deo S.K., Daunert S. (2017). Beyond Antibodies as Binding Partners: The Role of Antibody Mimetics in Bioanalysis. Annu. Rev. Anal. Chem..

[171] Kukowska-Latallo J.F., Candido K.A., Cao Z., Nigavekar S.S., Majoros I.J. (2005). Nanoparticle Targeting of Anticancer Drug Improves Therapeutic Response in Animal Model of Human Epithelial Cancer. Cancer Res..

[172] Bennewitz M.F., Saltzman W.M. (2009). Nanotechnology for Delivery of Drugs to the Brain for Epilepsy. Neurotherapeutics.

[173] Taratula O., Garbuzenko O.B., Kirkpatrick P., Pandya I., Savla R. (2009). Surface-Engineered Targeted PPI Dendrimer for Efficient Intracellular and Intratumoral siRNA Delivery. J. Control. Release.

[174] Zhu S., Hong M., Zhang L., Tang G., Jiang Y., Pei Y. (2010). PEGylated PAMAM Dendrimer-Doxorubicin Conjugates: In Vitro Evaluation and In Vivo Tumor Accumulation. Pharm. Res..

[175] Park J., Fong P.M., Lu J., Russell K.S., Booth C.J., Saltzman W.M., Fahmy T.M. (2009). PEGylated PLGA nanoparticles for the improved delivery of doxorubicin. Nanomedicine.

[176] Garcia-Garcia E., Andrieux K., Gil S., Couvreur P. (2005). Colloidal carriers and blood–brain barrier (BBB) translocation: A way to deliver drugs to the brain?. Int. J. Pharm..

[177] Chen D., Liu W., Shen Y., Mu H., Zhang Z. (2011). Effects of a novel pH-sensitive liposome with cleavable esterase-catalyzed and pH-responsive double smart mPEG lipid derivative on ABC phenomenon. Int. J. Nanomed..

[178] Martinho N., Damgé C., Reis C.P. (2011). Recent Advances in Drug Delivery Systems. J. Biomater. Nanobiotech..

[179] Song X., Zhao Y., Wu W., Bi Y., Cai Z. (2008). PLGA nanoparticles simultaneously loaded with vincristine sulfate and verapamil hydrochloride: Systematic study of particle size and drug entrapment efficiency. Int. J. Pharm..

[180] Ke W., Zhao Y., Huang R., Jiang C., Pei Y. (2008). Enhanced Oral Bioavailability of Doxorubicin in a Dendrimer Drug Delivery System. J. Pharmacol. Sci..

[181] Geldenhuys W., Mbimba T., Bui T., Harrison K., Sutariya V. (2011). Brain-targeted delivery of paclitaxel using glutathione-coated nanoparticles for brain cancers. J. Drug Target..

[182] Su C.W., Chiang C.S., Li W.M., Hu S.H., Chen S.Y. (2014). Multifunctional nanocarriers for simultaneous encapsulation of hydrophobic and hydrophilic drugs in cancer treatment. Nanomedicine.

[183] Hammady T., El-Gindy A., Lejmi E., Dhanikula R.S., Moreau P., Hildgen P. (2009). Characteristics and properties of nanospheres co-loaded with lipophilic and hydrophilic drug models. Int. J. Pharm..

[184] Amani A., Kabiri T., Shafiee S., Hamidi A. (2019). Preparation and Characterization of PLA-PEG-PLA/PEI/DNA Nanoparticles for Improvement of Transfection Efficiency and Controlled Release of DNA in Gene Delivery Systems. Iran. J. Pharm. Res..

[185] Van Vlerken L.E., Duan Z., Little S.R., Seiden M.V., Amiji M.M. (2008). Biodistribution and Pharmacokinetic Analysis of Paclitaxel and Ceramide Administered in Multifunctional Polymer-Blend Nanoparticles in Drug Resistant Breast Cancer Model. Mol. Pharm..

[186] Reis C.P., Neufeld R.J., Ribeiro A.J., Veiga F., Nanoencapsulation I. (2006). Methods for preparation of drug-loaded polymeric nanoparticles. Nanomedicine.

[187] Semete B., Booysen L., Lemmer Y., Kalombo L., Katata L. (2010). In vivo evaluation of the biodistribution and safety of PLGA nanoparticles as drug delivery systems. Nanomedicine.

[188] Fields C.J., Cheng E., Quijano C., Weller N., Kristofik N., Duong C., Hoimes M.E., Egan W.M. (2012). Saltzman, Surface modified poly(*β* amino ester)-containing nanoparticles for plasmid DNA delivery. J. Control. Release.

[189] Arvizo R.R., Miranda O.R., Moyano D.F., Wal-den C.A., Giri K. (2011). Modulating Pharmacokinetics, Tumor Uptake and Biodistribution by Engineered Nanoparticles. PLoS ONE.

[190] Testa B., Crivori P., Reist M., Pierre-Alain C. (2000). The influence of lipophilicity on the pharmacokinetic behavior of drugs: Concepts and examples. Perspect. Drug Discov. Des..

[191] Xin X.C., Nabisab M.M., Shaukat A.M., Abdul S.J., Awais A., Mohammad K., Rashmi W., Abdullah E.C., Rama R.K., Siddiqui M.T.H. (2021). A review on the properties and applications of chitosan, cellulose and deep eutectic solvent in green chemistry. J. Ind. Eng. Chem..

[192] Shoyaib A.A., Archie S.R., Karamyan V.T. (2020). Intraperitoneal Route of Drug Administration: Should it Be Used in Experimental Animal Studies?. Pharm. Res..

[193] Zheng W., Xue F., Zhang M., Wu Q., Yang Z., Ma S., Liang H., Wang C., Wang Y., Ai X. (2020). Charged Particle (Negative Ion)-Based Cloud Seeding and Rain Enhancement Trial Design and Implementation. Water.

[194] Zolnik B.S., González-Fernández Á., Sadrieh N., Dobrovolskaia M.A. (2010). Minireview: Nanoparticles and the Immune System. Endocrinology.

[195] Gustafson H.H., Holt-Casper D., Grainger D.W., Ghandehari H. (2015). Nanoparticle uptake: The phagocyte problem. Nano. Today.

[196] Sadekar S., Linares O., Noh G.J., Hubbard D., Ray A., Janát-Amsbury M., Peterson C.M., Facelli J., Ghandehari H. (2013). Comparative pharmacokinetics of PAMAM-OH dendrimers and HPMA copolymers in ovarian tumor-bearing mice. Drug Deliv. Transl. Res..

[197] Lee C.C., MacKay J.A., Fréchet J.M., Szoka F.C. (2005). Designing dendrimers for biological applications. Nat. Biotechnol..

[198] Chenthamara D., Subramaniam S., Ramakrishnan S.G., Krishnaswamy S., Essa M.M., Lin F.H., Qoronfleh M.W. (2019). Therapeutic efficacy of nanoparticles and routes of administration. Biomater. Res..

[199] Ryan G.M., Kaminskas L.M., Bulitta J.B., McIntosh M.P., Owen D.J., Porter C.J. (2013). EGylated polylysine dendrimers increase lymphatic exposure to doxorubicin when compared to PEGylated liposomal and solution formulations of doxorubicin. J. Control. Release.

[200] Nagai T. (1985). Adhesive topical drug delivery system. J. Control. Release.

[201] Yermak I.M., Davydova V.N., Volod’ko A.V. (2022). Mucoadhesive Marine Polysaccharides. Mar. Drugs.

[202] Keldibekova R., Suleimenova S., Nurgozhina G., Kopishev E. (2023). Interpolymer Complexes Based on Cellulose Ethers: Application. Polymers.

[203] Alvani M., Bahri Najafi R., Mahmood M., Fazel N. (2022). Preparation and pharmaceutical evaluation of Mucoadhesive Buccal film extracts of petroselinum for pharyngitis symptoms. Med. Sci. J. Islam. Azad Univesity-Tehran Med. Branch.

[204] Račić A., Krajišnik D. (2023). Biopolymers in Mucoadhesive Eye Drops for Treatment of Dry Eye and Allergic Conditions: Application and Perspectives. Pharmaceutics.

[205] Lee E., Park H.Y., Kim S.W., Sun Y., Choi J.H., Seo J., Jung Y.P., Kim A.J., Kim J., Lim K. (2023). Enhancing Supplemental Effects of Acute Natural Antioxidant Derived from Yeast Fermentation and Vitamin C on Sports Performance in Triathlon Athletes: A Randomized, Double-Blinded, Placebo-Controlled, Crossover Trial. Nutrients.

[206] Sharma P., Joshi R.V., Pritchard R., Xu K., Eicher M.A. (2023). Therapeutic Antibodies in Medicine. Molecules.

[207] Brito-Casillas Y., Caballero M.J., Hernández-Baraza L., Sánchez-Hernández R.M., Betancort-Acosta J.C., Wägner A.M. (2023). Ex vivo evaluation of adhesive strength and barrier effect of a novel treatment for esophagitis. Gastroenterol. Y Hepatol..

[208] Song S.Y., Ahn M.S., Mekapogu M., Jung J.A., Song H.Y., Lim S.H., Jin J.S., Kwon O.K. (2023). Analysis of Floral Scent and Volatile Profiles of Different Aster Species by E-nose and HS-SPME-GC-MS. Metabolites.

[209] Kali G., Fürst A., Efiana N.A., Dizdarević A., Bernkop-Schnürch A. (2023). Intraoral Drug Delivery: Highly Thiolated κ-Carrageenan as Mucoadhesive Excipient. Pharmaceutics.

[210] Voyutskii S.S., Vakula V.L. (1963). The role of diffusion phenomena in polymer-to-polymer adhesion. J. Appl. Polym. Sci..

[211] Gurny R., Meyer J.M., Peppas N.A. (1984). Bioadhesive intraoral release systems: Design, testing and analysis. Biomaterials.

[212] Minghao Z., Yuanyuan Q., Jinlong L., Lijun Y., Qingrong H., Xin J. (2023). Charge characteristics of guar gums on the three-stage interaction mechanism with mucin to improve the mucoadhesion ability. Food Hydrocoll..

[213] Nicholas A.P., Daniel A.C. (2009). Impact of Absorption and Transport on Intelligent Therapeutics and Nano-scale Delivery of Protein Therapeutic Agents. Chem. Eng. Sci..

[214] Mikos A.G., Peppas N.A. (1990). Bioadhesion: Possibilities and Future Trends: First International Joint Workshop of the Association for Pharmaceutical Technology (APV) and the Controlled Release Society (CRS).

[215] Morello G., De Iaco G., Gigli G., Polini A., Gervaso F. (2023). Chitosan and Pectin Hydrogels for Tissue Engineering and In Vitro Modeling. Gels.

[216] Georgiev N.I., Bakov V.V., Anichina K.K., Bojinov V.B. (2023). Fluorescent Probes as a Tool in Diagnostic and Drug Delivery Systems. Pharmaceuticals.

[217] Haimhoffer Á., Dossi E., Béresová M., Bácskay I., Váradi J., Afsar A., Rusznyák Á., Vasvári G., Fenyvesi F. (2021). Preformulation Studies and Bioavailability Enhancement of Curcumin with a ‘Two in One’ PEG-*β*-Cyclodextrin Polymer. Pharmaceutics.

[218] Jabbari E., Peppas N.A. (1999). Use of ATR-FTIR to study interdiffusion in polystyrene and poly(vinyl methyl ether). Macromolecules.

[219] Osorno L.L., Brandley A.N., Maldonado D.E., Yiantsos A., Mosley R.J., Byrne M.E. (2021). Review of Contemporary Self-Assembled Systems for the Controlled Delivery of Therapeutics in Medicine. Nanomaterials.

[220] De Gennes P.G. (1997). Soft Interfaces.

[221] Marques C., Leal-Júnior A., Kumar S. (2023). Multifunctional Integration of Optical Fibers and Nanomaterials for Aircraft Systems. Materials.

[222] Huang Y., Szleifer I., Peppas N.A. (2001). Gel–gel adhesion by tethered polymers. J. Chem. Phys..

[223] Riley R.G., Green K.L., Smart J.D. (2001). The gastrointestinal transit profile of ^14^C-labelled poly(acrylic acids): An in vivo study. Biomaterials.

[224] Lowman A.M., Peppas N.A. (1999). Hydrogels, Encyclopedia of Controlled Drug Delivery.

[225] Peppas N.A., Huang Y., Torres-Lugo M., Ward J.H., Zhang J. (2000). Physicochemical foundations and structural design of hydrogels in medicine and biology. Annu. Rev. Biomed. Eng..

[226] Peppas N.A., Bures P., Leobandung W., Ichikawa H. (2000). Hydrogels in pharmaceutical formulations. J. Pharm. Biopharm..

[227] Dziubla T.D., Lowman A.M., Peppas N.A. (2001). Evaluation of Poly(ethylene glycol)-Based Copolymers for Contact Lenses. Trans. Soc. Biomater..

[228] Peppas N.A., Ratner B.D., Hoffman A.S., Schoen F.J., Lemons J.E. (2004). Biomaterials Science: An Introduction to Materials in Medicine.

[229] Peppas N.A., Langer R. (1994). New challenges in biomaterials. Science.

[230] Park K. (1997). Controlled Drug Delivery: Challenges and Strategies.

[231] Brannon-Peppas L., Peppas N.A. (2002). Polymer Science of Controlled Release Systems.

[232] Peppas N.A., Wood K.M., Blanchette J.O. (2004). Hydrogels for oral delivery of therapeutic proteins. Biol. Ther..

[233] Peppas N.A., Zhang J. (2000). Diffusional Behavior in pH- and Temperature Sensitive Interpenetrating Polymeric Networks Used in Drug Delivery, Biomaterials and Drug Delivery Systems towards the New Millennium.

[234] Peppas N.A. (2004). Kinetics of Smart Hydrogels, Reflexive Polymers and Hydrogels: Understanding and Designing Fast-Responsive Polymeric Systems.

[235] Horue M., Silva J.M., Berti I.R., Brandão L.R., Barud H.D.S., Castro G.R. (2023). Bacterial cellulose-based materials as dressings for wound healing. Pharmaceutics.

[236] Ciolacu D.E., Nicu R., Suflet D.M., Rusu D., Darie-Nita R.N., Simionescu N., Ciolacu F. (2023). Multifunctional Hydrogels Based on Cellulose and Modified Lignin for Advanced Wounds Management. Pharmaceutics.

[237] Lowman A.M., Morishita M., Kajita M., Nagai T., Peppas N.A. (1999). Oral delivery of insulin using pH-responsive complexation gels. J. Pharm. Sci..

[238] Foss A.C., Goto T., Morishita M., Peppas N.A. (2004). Development of acrylic-based copolymers for oral insulin delivery. J. Pharm. Biopharm..

[239] Torres-Lugo M., Garcia M., Record R., Peppas N.A. (2002). pH-Sensitive Hydrogels as Gastrointestinal Tract Absorption Enhancers: Transport Mechanisms of Salmon Calcitonin and Other Model Molecules Using the Caco-2 Cell Model. Biotechnol. Prog..

[240] Lowman A.M., Peppas N.A. (2000). Molecular analysis of interpolymer complexation in graft copolymer networks. Polymer.

[241] Kim B., Peppas N.A. (2003). Analysis of molecular interactions in poly(methacrylicacid-*g*-ethyleneglycol) hydrogels. Polymer.

[242] Lowman A.M. (2000). Complexing Polymers in Drug Delivery, Handbook of Pharmaceutical Controlled Release Technology.

[243] Narasimhan B., Peppas N.A. (1997). The Role of Modeling Studies in the Development of Future Controlled Release Devices, Controlled Drug Delivery: Challenges and Strategies.

[244] Heller J. (1987). Use of Polymers in Controlled Release of Active Agents, Controlled Drug Delivery, Fundamentals and Applications.

[245] Burnette R.R. (1987). Theory of Mass Transfer, Controlled Drug Delivery, Fundamentals and Applications.

[246] Ritger P.L., Peppas N.A. (1987). A simple equation for description of solute release I. Fickian and non-fickian release from non-swellable devices in the form of slabs, spheres, cylinders or discs. J. Control. Release.

[247] Fonseka P., Marzan A.L., Mathivanan S., Mathivanan S., Fonseka P., Nedeva C., Atukorala I. (2021). Introduction to the Community of Extracellular Vesicles. New Frontiers: Extracellular Vesicles.

[248] Rai A., Fang H., Fatmous M., Claridge B., Poh Q.H., Simpson R.J., Greening D.W. (2021). A Protocol for Isolation, Purification, Characterization, and Functional Dissection of Exosomes. Methods Mol. Biol..

[249] Garcia-Martin R., Wang G., Brandão B.B., Zanotto T.M., Shah S., Kumar Patel S., Schilling B., Kahn C.R. (2022). MicroRNA Sequence Codes for Small Extracellular Vesicle Release and Cellular Retention. Nature.

[250] Mallick S.P., Singh B.N., Rastogi A., Srivastava P. (2018). Design and Evaluation of Chitosan/Poly(l-Lactide)/Pectin Based Composite Scaffolds for Cartilage Tissue Regeneration. Int. J. Biol. Macromol..

[251] Zhang Y., Zhang P., Gao X., Chang L., Chen Z., Mei X. (2021). Preparation of Exosomes Encapsulated Nanohydrogel for Accelerating Wound Healing of Diabetic Rats by Promoting Angiogenesis. Mater. Sci. Eng. C.

[252] Yang Z., Yang Y., Xu Y., Jiang W., Shao Y., Xing J., Chen Y., Han Y. (2021). Biomimetic Nerve Guidance Conduit Containing Engineered Exosomes of Adipose-Derived Stem Cells Promotes Peripheral Nerve Regeneration. Stem Cell Res. Ther..

[253] Lv K., Li Q., Zhang L., Wang Y., Zhong Z., Zhao J., Lin X., Wang J., Zhu K., Xiao C. (2019). Incorporation of Small Extracellular Vesicles in Sodium Alginate Hydrogel as a Novel Therapeutic Strategy for Myocardial Infarction. Theranostics.

[254] Cunnane E.M., Lorentz K.L., Ramaswamy A.K., Gupta P., Mandal B.B., O’Brien F.J., Weinbaum J.S., Vorp D.A. (2020). Extracellular Vesicles Enhance the Remodeling of Cell-Free Silk Vascular Scaffolds in Rat Aortae. ACS Appl. Mater. Interfaces.

[255] Wu D., Qin H., Wang Z., Yu M., Liu Z., Peng H., Liang L., Zhang C., Wei X. (2022). Bone Mesenchymal Stem Cell-Derived SEV-Encapsulated Thermosensitive Hydrogels Accelerate Osteogenesis and Angiogenesis by Release of Exosomal MiR-21. Front. Bioeng. Biotechnol..

[256] Xin L., Lin X., Zhou F., Li C., Wang X., Yu H., Pan Y., Fei H., Ma L., Zhang S. (2020). A Scaffold Laden with Mesenchymal Stem Cell-Derived Exosomes for Promoting Endometrium Regeneration and Fertility Restoration through Macrophage Immunomodulation. Acta Biomater..

[257] Man K., Barroso I.A., Brunet M.Y., Peacock B., Federici A.S., Hoey D.A., Cox S.C. (2022). Controlled Release of Epigenetically-Enhanced Extracellular Vesicles from a GelMA/Nanoclay Composite Hydrogel to Promote Bone Repair. Int. J. Mol. Sci..

[258] Nikhil A., Kumar A. (2022). Evaluating Potential of Tissue-Engineered Cryogels and Chondrocyte Derived Exosomes in Articular Cartilage Repair. Biotechnol. Bioeng..

[259] Kwak G., Cheng J., Kim H., Song S., Lee S.J., Yang Y., Jeong J.H., Lee J.E., Messersmith P.B., Kim S.H. (2022). Sustained Exosome-Guided Macrophage Polarization Using Hydrolytically Degradable PEG Hydrogels for Cutaneous Wound Healing: Identification of Key Proteins and MiRNAs, and Sustained Release Formulation. Small.

[260] Wei Y., Wu Y., Zhao R., Zhang K., Midgley A.C., Kong D., Li Z., Zhao Q. (2019). MSC-Derived SEVs Enhance Patency and Inhibit Calcification of Synthetic Vascular Grafts by Immunomodulation in a Rat Model of Hyperlipidemia. Biomaterials.

[261] Ko K.-W., Park S.-Y., Lee E.H., Yoo Y.-I., Kim D.-S., Kim J.Y., Kwon T.G., Han D.K. (2021). Integrated Bioactive Scaffold with Polydeoxyribonucleotide and Stem-Cell-Derived Extracellular Vesicles for Kidney Regeneration. ACS Nano.

[262] Swanson W.B., Zhang Z., Xiu K., Gong T., Eberle M., Wang Z., Ma P.X. (2020). Scaffolds with Controlled Release of Pro-Mineralization Exosomes to Promote Craniofacial Bone Healing without Cell Transplantation. Acta Biomater..

[263] Gandolfi M.G., Gardin C., Zamparini F., Ferroni L., Esposti M.D., Parchi G., Ercan B., Manzoli L., Fava F., Fabbri P. (2020). Mineral-Doped Poly(L-Lactide) Acid Scaffolds Enriched with Exosomes Improve Osteogenic Commitment of Human Adipose-Derived Mesenchymal Stem Cells. Nanomaterials.

[264] Leung K.S., Shirazi S., Cooper L.F., Ravindran S. (2022). Biomaterials and Extracellular Vesicle Delivery: Current Status, Applications and Challenges. Cells.

[265] Sridhar R., Ramakrishna S. (2013). Electrosprayed nanoparticles for drug delivery and pharmaceutical applications. Biomatter..

[266] Kinoshita M. (2006). Targeted Drug Delivery to the Brain Using Focused Ultrasound. Top. Magn. Reson. Imaging.

[267] Jones A.R., Shusta E.V. (2007). Blood-brain barrier transport of therapeutics via receptor-mediation. Pharma. Res..

[268] Dadparvar M., Wagner S., Wien S., Kufleitner J., Worek F., Von Briesen H., Kreuter J. (2011). HI 6 human serum albumin nanoparticles-development and transport over an in vitro blood-brain barrier model. Toxicol. Lett..

[269] Tiwari G., Tiwari R., Rai A.K. (2010). Cyclodextrins in delivery systems: Applications. J. Pharm. Bioallied Sci..

[270] Zuccari G., Alfei S. (2023). Development of Phytochemical Delivery Systems by Nano-Suspension and Nano-Emulsion Techniques. Int. J. Mol. Sci..

[271] Hussein N.R., Omer H.K., Abdelbary M.A.E., Ahmed W. (2020). Chapter 15—Advances in nasal drug delivery systems. Advances in Medical and Surgical Engineering.

[272] Hall D.J., Khutoryanskaya O.V., Khutoryanskiy V.V. (2011). Developing synthetic mucosa-mimetic hydrogels to replace animal experimentation in characterization of mucoadhesive drug delivery systems. Soft Matter.

[273] Zhu Q., Talton J., Zhang G., Cunningham T., Wang Z., Waters R.C., Kirk J., Eppler B., Klinman D.M., Sui Y. (2012). Large intestine-targeted, nanoparticle-releasing oral vaccine to control genitorectal viral infection. Nat. Med..

[274] Lawson L.B., Norton E.B., Clements J.D. (2011). Defending the mucosa: Adjuvant and carrier formulations for mucosal immunity. Curr. Opin. Immunol..

[275] Sandri S.R.G., Bonferoni M.C., Ferrari F., Mori M., Caramella C. (2012). The role of chitosan as a mucoadhesive agent in mucosal drug delivery. J. Drug Deliv. Sci. Technol..

[276] Lam J.K.W., Cheung C.C.K., Chow M.Y.T., Harrop E., Lapwood S., Barclay S.I.G., Wong I.C.K. (2020). Transmucosal drug administration as an alternative route in palliative and end-of-life care during the COVID-19 pandemic. Adv. Drug Deliv. Rev..

[277] Wang C., Chu C., Ji X., Luo G., Xu C., He H., Yao J., Wu J., Hu J., Jin Y. (2022). Biology of Peptide Transporter 2 in Mammals: New Insights into Its Function Structure and Regulation. Cells.

[278] Mikihisa T., Shiori K., Nanako K., Masashi K., Ryoko Y. (2022). Effect of Corticosteroids on Peptide Transporter 2 Function and Induction of Innate Immune Response by Bacterial Peptides in Alveolar Epithelial Cells. Biol. Pharm. Bull..

[279] Banat H., Ambrus R., Csóka I. (2023). Drug combinations for inhalation: Current products and future development addressing disease control and patient compliance. Int. J. Pharm..

[280] Smola M., Vandamme T., Sokolowski A. (2008). Nanocarriers as pulmonary drug delivery systems to treat and to diagnose respiratory and non-respiratory diseases. Int. J. Nanomed..

[281] Czajkowska-Kosnik A., Szekalska M., Winnicka K. (2019). Nanostructured lipid carriers: A potential use for skin drug delivery systems. Pharmacol. Rep..

[282] Brown M.B., Martin G.P., Jones S.A., Akomeah F.K. (2006). Dermal and transdermal drug delivery systems: Current and future prospects. Drug Deliv..

[283] Clinical Trials.Gov Website. https://classic.clinicaltrials.gov/.

[284] Pandit A., Zeugolis D.I. (2016). Twenty-five years of nano-bio-materials: Have we revolutionized healthcare?. Nanomedicine.

